# Restoration of dysregulated intestinal barrier and inflammatory regulation through synergistically ameliorating hypoxia and scavenging reactive oxygen species using ceria nanozymes in ulcerative colitis

**DOI:** 10.1186/s40824-023-00412-8

**Published:** 2023-07-28

**Authors:** Ying Zhang, Hengyu Lei, Pengchong Wang, Qinyuan Zhou, Jie Yu, Xue Leng, Ruirui Ma, Danyang Wang, Kai Dong, Jianfeng Xing, Yalin Dong

**Affiliations:** 1 Department of Pharmacy, The First Affiliated Hospital of Xi’an Jiaotong University, Xi’an, Shaanxi, 710061 China; 2 Department of Pharmaceutics, School of Pharmacy, Xi’an Jiaotong University, Xi’an, Shaanxi, 710061 China; 3 Department of Pharmacy, Shaanxi Provincial People’s Hospital, Xi’an, Shaanxi, China

**Keywords:** Ceria nanozymes, Ulcerative colitis, Hypoxia-inducible factor-1α, Reactive oxygen species, Inflammatory regulation

## Abstract

**Background:**

Reactive oxygen species (ROS) overproduction and excessive hypoxia play pivotal roles in the initiation and progression of ulcerative colitis (UC). Synergistic ROS scavenging and generating O_2_ could be a promising strategy for UC treatment.

**Methods:**

Ceria nanozymes (PEG-CNPs) are fabricated using a modified reverse micelle method. We investigate hypoxia attenuating and ROS scavenging of PEG-CNPs in intestinal epithelial cells and RAW 264.7 macrophages and their effects on pro-inflammatory macrophages activation. Subsequently, we investigate the biodistribution, pharmacokinetic properties and long-term toxicity of PEG-CNPs in mice. PEG-CNPs are administered intravenously to mice with 2,4,6-trinitrobenzenesulfonic acid-induced colitis to test their colonic tissue targeting and assess their anti-inflammatory activity and mucosal healing properties in UC.

**Results:**

PEG-CNPs exhibit multi-enzymatic activity that can scavenge ROS and generate O_2_, promote intestinal epithelial cell healing and inhibit pro-inflammatory macrophage activation, and have good biocompatibility. After intravenous administration of PEG-CNPs to colitis mice, they can enrich at the site of colonic inflammation, and reduce hypoxia-induced factor-1α expression in intestinal epithelial cells by scavenging ROS to generate O_2_, thus further promoting disrupted intestinal mucosal barrier restoration. Meanwhile, PEG-CNPs can effectively scavenge ROS in impaired colon tissues and relieve colonic macrophage hypoxia to suppress the pro-inflammatory macrophages activation, thereby preventing UC occurrence and development.

**Conclusion:**

This study has provided a paradigm to utilize metallic nanozymes, and suggests that further materials engineering investigations could yield a facile method based on the pathological characteristics of UC for clinically managing UC.

**Graphical Abstract:**

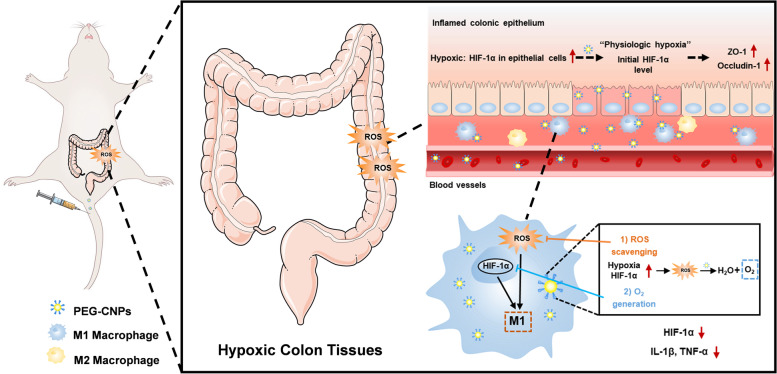

**Supplementary Information:**

The online version contains supplementary material available at 10.1186/s40824-023-00412-8.

## Introduction

Ulcerative colitis (UC) is a chronic non-specific inflammatory disease characterized by chronic inflammation of the colon [1]. The course of UC is protracted and recurrent, which seriously affects the health and life quality of patients and increases the risk of colitis-associated cancer [2]. The pathogenesis of UC is associated with disrupted intestinal barrier functions, dysregulated mucosal immune response and imbalance of the gut microbiome [3]. Traditional treatments (using anti-inflammatory, immunomodulatory agents) for UC are mainly restricted to control inflammation [4]; however, they usually ignore the procrastination and recurrence of UC caused by the mucosal damage and dysregulated intestinal barrier functions [5]. Moreover, long-term use of aminosalicylic acids, glucocorticoids and immunosuppressive agents may lead to severe complications, such as malignant tumors, autoimmunity and hepatotoxicity [4, 6]. Therefore, reducing local intestinal inflammatory response and restoring damaged intestinal mucosal barriers is the key to the treatment of UC.

Reactive oxygen species (ROS), which are by-products of aerobic metabolism, are crucial molecules in physiological processes [7]. However, their overproduction in the intestine will cause oxidative stress that will lead to intestinal endothelial cell damage through inducing lipid peroxidation and DNA mutation, impairing protein functions, altering epithelial permeability, and disrupting intestinal epithelial barriers, eventually leading to the initiation or deterioration of UC [8, 9]. Furthermore, excessive ROS can also cause dysregulated pro-inflammatory reactive species-sensitive pathways in immune cells [10]. Nuclear factor-erythroid 2-related factor 2 (Nrf2) is a redox-sensitive transcription factor [11]. Activation of Nrf2 can attenuate inflammatory damage and neutralize ROS of cells/tissues from inflammatory injuries [12]. Recent studies have demonstrated that activation of Nrf2 can protect against dextran sulfate sodium (DSS)-induced colitis and inflammation-associated colorectal cancer by maintenance of intestinal integrity and regulation of pro-inflammatory cytokines [13, 14].

In addition to excessive ROS in the colon tissue, UC is associated with infiltration of macrophage in the colonic inflammatory tissue [15]. Pro-inflammatory macrophages are predominant during colon inflammation, and excessively infiltrate at the inflammatory sites of the colon and continuously release inflammatory chemokines and pro-inflammatory cytokines, thereby eliciting an inflammatory cascade response that leads to a continuous vicious cycle of inflammation [16, 17]. At the inflammatory site of colon mucosa, local depletion of nutrients, imbalance in tissue oxygen supply and demand, and the generation of large quantities of reactive nitrogen and oxygen intermediates lead to aggravating hypoxia in the intestinal epithelial cell layer. Pro-inflammatory macrophages have been shown to select metabolic pathways relying on glycolysis for energy, which suggests that hypoxia drives pro-inflammatory macrophage polarization and promotes pro-inflammatory factor secretion [18, 19]. Hypoxia-inducible factor-1α (HIF-1α) transcription factor is the master regulator of the cellular response to hypoxia [20, 21], a microenvironmental feature of site of colon inflammation [22]. In colon inflammatory tissues, in addition to hypoxia, HIF-1α can be activated via the nuclear factor-kappa B (NF-κB) [23]. It is shown that HIF-1α overexpression induces NF-κB-regulated inflammatory cytokine expression as well as mediates NF-κB activation in hypoxic neutrophils [24, 25]. Moreover, HIF-1α overexpression induces changes in mitochondrial structure, dynamics, and genome stability, resulting in decreased mitochondrial respiration, increased oxidative damage, and excessive reactive oxygen species (ROS) production [26, 27]. For UC, hypoxia of macrophages and the overproduction of ROS at the inflammation site can aggravate the colon inflammation and induce the polarization of pro-inflammatory macrophages [22, 28–31]. Simultaneously ameliorating hypoxia of macrophages and decreasing ROS levels to inhibit the pro-inflammatory macrophages activation might therefore be a promising strategy for UC treatment.

Effective epithelial barrier is important in intestinal homeostasis, and barrier dysfunction underpins inflammatory bowel disease [32]. As a result of this unique anatomy, intestinal epithelial cells function in a steep physiological oxygen gradient relative to other cell types, known as “physiological hypoxia”. Under physiological conditions, intestinal epithelial cells exposed to hypoxia can protect barrier function and reduce the inflammatory response by up-regulating intestinal trilobin factor, mucin-3 and CD73 through stabilization of HIF-1α expression [33, 34]. However, homeostasis destruction in the “physiological hypoxia” area of ​​the intestine promotes the occurrence and development of colitis [35]. At the inflammatory site of colon mucosa, imbalance of O_2_ supply leads to hypoxia aggravation in the intestinal epithelial cell layer, which lead to HIF-1α overexpression. HIF-1α overexpression can subsequently stimulate tumor necrosis factor secretion in the intestinal epithelial cells, thereby increasing barrier permeability and exacerbating disease conditions, leading to intestinal damage, inflammation, and colorectal cancer [36]. Hence, during the occurrence and development of UC, ameliorating excessive hypoxia in epithelial cells through oxygen generation can help promote restoration of intestinal mucosal barriers, relieve the symptoms of UC and reduce the recurrence of UC.

Nanomaterials with catalase (CAT)-mimicking and/or superoxide dismutase (SOD)-mimicking activities, such as ceria nanozymes (CNPs) [37], copper-based nanoparticles [38], molybdenum-based nanoparticles [39], manganese-based nanoparticles [40, 41] and Prussian blue nanoparticles [42], have been widely investigated in ROS-related inflammatory diseases [43–45]. More importantly, due to the CAT-mimicking activity of these nanomaterials, they can generate oxygen during ROS scavenging process, which is beneficial for alleviating inflammation [46]. In particular, owing to the coexistence of Ce^3+^ (reduced) and Ce^4+^ (oxidized) at the particle surface, CNPs with excellent ROS scavenging capacity have been applied in biomedical applications, including inflammatory diseases [47, 48], nervous system disease [49–51], wound healing [52, 53] and so on. Although it has been reported the therapeutic effect of CNPs on experimental colitis [54], the mechanism responsible for anti-UC effect of CNPs, such as the therapeutic effect of O_2_ production during ROS scavenging, the inhibition of pro-inflammatory macrophages polarization and the protective effect on intestinal epithelial cells, has not been reported. An in-depth study of the mechanism of the anti-UC effect of CNPs is of great significance for the application of CNPs and such nanozymes in the treatment of UC.

**Scheme 1:**
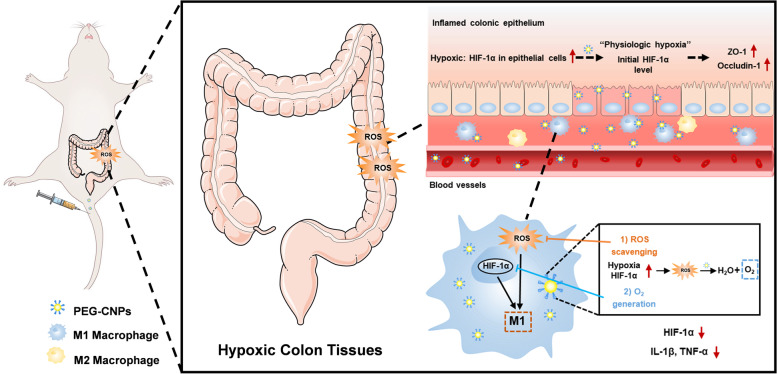
Restoration of dysregulated intestinal barrier and inflammatory regulation using ceria nanozymes for the treatment of ulcerative colitis. The scheme of therapeutic mechanism of PEG-CNPs for synergistically ameliorating hypoxia and scavenging reactive oxygen species with superoxide dismutase/catalase-mimicking activity

Herein we synthesized PEG-CNPs using a modified reverse micelle method and uncovered dual regulatory roles of PEG-CNPs for managing UC: (1) generating O_2_ and scavenging ROS in damaged intestinal epithelial cells, thereby promoting colonic epithelium restoration, and (2) alleviating colon macrophage hypoxia for inflammatory regulation to prevent UC occurrence and development (Scheme 1). We further investigated the mechanisms underlying how PEG-CNPs act against UC, which included the influences on the expression of intracellular HIF-1α, level of pro-/anti-oxidation gene, and pro-inflammatory macrophages activation. Our study provides a proof of concept for PEG-CNPs as a safe and potentially effective nanomedicine for clinical UC management.

## Materials and methods

### Materials

Cerium acetate (CAS: 206,996–60-3, Lot: E1822034, 99.99%) was obtained from Aladdin Industrial Inc. (Shanghai, China). Oleylamine (CAS: 112–90-3, Lot: C14418632, 80–90%) was obtained from Macklin Biochemical Co., Ltd (Shanghai, China). Xylene (CAS: 1330–20-7, Lot: 74,847,021, 99%) was obtained from Meryer Technologies Co., Ltd. (Shanghai, China). 1,2-distearoyl-sn-glycero-3-phosphoethanolamine-N-[methoxy (polyethylene glycol)-2000] (DSPE-mPEG_2000_ (CAS: 147,867–65-0, Lot: 9JEDMRAQ, ≥ 95%) was purchased from Alab (Shanghai) Chemical Technology Co., Ltd. DSPE-mPEG_2000_-FITC was obtained from Xi’an Ruixi Biological Technology Co., Ltd. Hydrogen peroxide (H_2_O_2_) was purchased from Sinopharm Chemical Reagent Co., Ltd. (Shaanxi, China).

### Animals

Specific pathogen-free male ICR mice (23–25 g) were purchased from Laboratory Animal Center of Xi’an Jiaotong University. Animals were supplied with normal chow and water ad libitum at a temperature of 22–25 °C with a 12 h light–dark cycle and 65 ± 5% humidity. All work performed on animals was in strict accordance with the Guidelines of the Laboratory Animal Center of Xi’an Jiaotong University and approved by the Institutional Animal Care and Use Committee of Xi’an Jiaotong University (No. XJTULAC2019-068).

### Synthesis of ceria nanoparticles (OA-CNPs)

Ceria nanoparticles were synthesized as previously described [55]. Firstly, 0.43 g cerium acetate and 3.25 g oleylamine were added to 15 mL of xylene. The resulting solution was stirred at room temperature for 2 h and then slowly heated to 90 °C under nitrogen environment. One milliliter of deionized water was injected into the mixture under vigorous stirring to initiate a sol–gel reaction. After aging the mixture at 90 °C for 3 h and the color changed into a pale-yellow colloidal solution, cooling the mixture to room temperature. A total of 100 mL of ethanol was added to the precipitated ceria nanoparticles. After precipitation and washed by ethanol for three times, the OA-CNPs were dispersed in chloroform for further experiments.

### Synthesis of phospholipid-PEG modified ceria nanoparticles (PEG-CNPs)

5 mg OA-CNPs were mixed with 10 mL chloroform containing 25 mg DSPE-mPEG_2000_ and sonicated for 10 min. Then, solvents were evaporated by rotary evaporation under vacuum for 1 h at 60 °C. Five milliliter of deionized water was added to re-disperse the dried mixture to obtain the DSPE-mPEG_2000_ modified PEG-CNPs. FITC-PEG-CNPs were synthesis by the same procedures of PEG-CNPs, except replacing 25 mg DSPE-mPEG_2000_ with 20 mg DSPE-mPEG_2000_ and 5 mg DSPE-mPEG_2000_-FITC.

### Characterization

Transmission electron microscopy (TEM) analysis was performed to observe the morphology of PEG-CNPs (JEOL JEM-2100Plus, Japan) at 100 kV. High resolution transmission electron microscopy (HRTEM) was taken with a FEI Tecnai F20 (FEI, USA) at a voltage of 200 kV. The hydrodynamic size and zeta potential of PEG-CNPs were detected using Zetasizer Nano ZS90 (Malvern Instruments, UK). The X-ray powder diffraction (XRD) pattern was detected on an XRD-6100 (Shimadzu, Japan). The X-ray photoelectron spectra (XPS) was acquired via a Thermo Scientific ESCALAB 250 Xi XPS system. The concentration of the cerium element was quantified using the Inductively coupled plasma-Mass Spectrometry (ICP-MS, PerkinElmer NexION 350D).

### CAT-mimicking activity of PEG-CNPs

Different concentrations of PEG-CNPs (0.1 mM, 0.2 mM, 0.4 mM and 0.8 mM) and 1 M H_2_O_2_ were added into deionized water. The generated oxygen concentration within 30 min was evaluated by an oxygen electrode on Dissolved Oxygen Meter JPB-607 (Shanghai INESA Scientific Instrument, China).

### SOD-mimicking activity of PEG-CNPs

The SOD-mimicking activity of PEG-CNPs at different concentrations (0.1 mM, 0.2 mM, 0.4 mM and 0.8 mM) was measured by a Total Superoxide Dismutase Assay Kit with WST-8 (Beyotime, Shanghai, China).

### Hydroxyl radical antioxidant capacity of PEG-CNPs

The ·OH scavenging activity of PEG-CNPs at different concentrations (0.1 mM, 0.2 mM, 0.4 mM and 0.8 mM) was measured by a Hydroxyl Free Radical Assay Kit (Nanjing Jiancheng Bioengineering Institute, China).

### Cell culture

Human colonic adenocarcinoma (HT-29) cells and RAW 264.7 cells were purchased from Cell Bank of the China Science Academy (Shanghai, China). HT-29 cells are grown in McCoy’s 5A medium (Gibco, Thermo Fisher, Massachusetts, USA) containing 10% fetal bovine serum (Gibco). RAW 264.7 cells are grown in DMEM medium (Gibco, Thermo Fisher, Massachusetts, USA) containing 10% fetal bovine serum (Gibco).

### Cell uptake

HT-29 cells or RAW 264.7 cells were seeded on cell slides with a glass-bottom insert and incubated for 24 h. Then 50 μM FITC-PEG-CNPs were added and the cells were incubated at 37 °C for 24 h. The cells were then washed with PBS and observed by a fluorescent microscopy (Carl Zeiss, Jena, Germany) to confirm the cellular uptake.

### Cell viability assay

CCK-8 assay was used to evaluate in vitro cytotoxicity. To test the cytotoxicity of various concentrations of PEG-CNPs, HT-29 and RAW 264.7 cells were seeded in 96-well plates at a density of 0.5 × 10^4^ cells/well and cultured in 5% CO_2_ at 37°C for 24 h. Then, PEG-CNPs were added to the wells. At the end of the incubation, the medium was removed, and 100 μL of fresh medium and 10 μL of CCK-8 solution were added and incubated for another 1 h, and the absorbance was monitored using a microplate reader at the wavelength of 450 nm. Furthermore, before the treatment of H_2_O_2_ in HT-29 cells, various concentrations of PEG-CNPs were added and the cell viability was investigated by CCK-8.

### Intracellular O_2_ level measurement with O_2_ indicator [Ru (dpp)_3_].^2+^Cl_2_ {tris (4, 7-diphenyl-1,10-phenanthroline) ruthenium(II) dichloride}

PEG-CNPs (50 μM) were incubated with hypoxia RAW 264.7 cells (1% O_2_, 5% CO_2_, and 94% N_2_ gas for 4 h). The O_2_ indicator [Ru(dpp)_3_]^2+^Cl_2_ was added, with the final concentration of 2 μM. Then the intracellular fluorescence was observed by fluorescent microscopy.

### Intracellular H_2_O_2_ level measurement with H_2_O_2_ indicator DCFH-DA (2’, 7’-dichlorodihydrofluorescein diacetate)

ROS scavenging ability of PEG-CNPs was investigated by DCFH-DA assay. HT-29 cells and RAW 264.7 cells were cultured in 6-well plates, respectively. After HT-29 cells or RAW 264.7 cells with 50 µM PEG-CNPs treatment for 2 h, the cells were treated with 10 mM H_2_O_2_ for 12 h, or 50 μg/ml Rosup for 1 h, respectively. Then, the cells were washed with PBS and incubated with 10 µM DCFH-DA for 30 min under dark environment. Then the intracellular fluorescence was observed by fluorescent microscopy.

### Morphological of RAW 264.7 cells

RAW 264.7 cells were treated with 50 μM PEG-CNPs for 2 h after LPS (-/ +) (100 ng/mL) treatment and the images were taken at 24 h after LPS treatment by using a LEICA DMI 4000B microscope.

### Flow cytometry for macrophage polarization

RAW 264.7 cells were treated with 50 μM PEG-CNPs for 2 h after LPS (-/ +) (100 ng/mL) treatment. Then, RAW 264.7 cells were collected at 24 h and were incubated with Tru Stain FcX™ (anti-mouse CD16/32), and then stained with the following antibodies: CD86 (PE, Biolegend, 207,219). After staining, all cells were subjected to intracellular staining perm wash buffer and analyzed in a BD FACS-Calibur cytometer (Becton Dickinson, San Jose, CA).

### Cell apoptosis assay

HT-29 cells were treated with 50 µM PEG-CNPs for 2 h after H_2_O_2_ treatment. Annexin V-FITC/7-AAD Apoptosis Detection Kit (Procell, China) was used to statin the cells after 24 h and cell apoptosis rate was analyzed via flow cytometry.

### Cell scratch test

HT-29 cells were cultured in 6-well plates for 24 h. After cells reached absolute confluence, 10 μL sterilized pipette tip was used to create parallel scratches. Then, the cells were washed with PBS, and serum-free medium with PEG-CNPs solution was added under hypoxic condition. The cell migration was observed using microscope.

### RNA extraction and qRT-PCR

Total RNA was isolated from RAW 264.7 cells and HT-29 cells by using TRIzol reagent (Invitrogen, Carlsbad, California, USA). The concentration of total RNA was measured using NanoDrop spectrometer (ND-2000, Thermo Scientific, USA). qRT-PCR was performed on CFX96 Real-Time PCR Detection System (Bio-Rad, Hercules, California, USA) using TB Green Premix Ex Tag II (TaKaRa Bio Inc., Japan) with 25 μL reaction mixtures (primers are enlisted in Table S1). PCR program was reacted with amplification with 39 cycles at 95°C for 30 s, at 95°C for 5 s, and at 60°C for 30s. The relative mRNA levels were quantified by the 2^−ΔΔCt^ method and all data were normalized to GAPDH (the internal control). The sequences of all primers involving in this study were listed in Table S1.

### ELISA

RAW 264.7 cells were treated with 50 μM PEG-CNPs for 2 h after LPS (-/ +) (100 ng/mL) treatment for 24 h. Culture supernatants were collected and preserved at -80℃. The expression of inflammatory cytokines (TNF-α, IL-1β, IL-10 and TGF-β1) in culture supernatants were measured by ELISA kits (Beyotime, China) according to the kit instructions.

### Western blot

Immunoblotting analysis of proteins in HT-29 cells and RAW 264.7 cells was performed in the lysis buffer containing sodium dodecyl sulfate lysis buffer (Beyotime, Shanghai, China) and protease and phosphatase inhibitor cocktail (Thermo Fisher, USA) for 30 min. The protein concentration was assessed with a BCA assay kit (Beyotime, Shanghai, China). Protein extracts were separated on sodium dodecyl sulfatepolyacrylamide gel electrophoresis (8–12% gels) and blotted onto PVDF membranes (Merck Millipore Ltd., Massachusetts, USA). After blocking with 5% fat-free milk, the membranes were incubated overnight at 4 °C with the following primary antibodies: HIF-1α (1:1000, ab179483), and Actin (1:1000, ab8226). The binding antibodies were detected by horseradish peroxidase-conjugated IgG (Multi Sciences) and visualized with enhanced chemiluminescence detection reagents (FDbio Science Biotech Co., Ltd., Hangzhou, China).

### Nrf2 knockdown by small interfering RNA (siRNA)

To silence Nrf2 gene expression, Nrf2-specific siRNA (Si*Nrf2*) and control siRNA (SiNC) were obtained from Genepharma (Shanghai, China). HT-29 cells were transfected with siRNA (Si*Nrf2* or SiNC) and Lipofectamine 2000 transfection reagent. The medium (OptiMEM) was replaced at 6 h after transfection. After 24 h, the cells were treated with H_2_O_2_ and PEG-CNPs. Si*Nrf2* sequences were listed in Table S1.

### Safety evaluation

The safety of intravenous injection was first examined by a hemolysis study. The concentrations of PEG-CNPs were 1, 2, 5, and 10 μM. The negative control was given PBS; and the positive control was given deionized water. For each group, 2 mL of erythrocyte suspension was taken in a 15 mL centrifuge tube. Then 2 mL of sample was added separately, bathed in water bath at 37 °C for 4 h, and centrifuge at 1000 rpm/min for 5 min. Simultaneously, 1 mL of supernatant was taken from each group, and the absorbance was measured by UV–vis at 540 nm.

PEG-CNPs (1.5 mg/kg) or normal saline were intravenously injected into healthy mice were used as the PEG-CNPs group and control group, respectively (*n* = 6). Animals were sacrificed and the major organs (kidney, lung, spleen, liver, and heart) and serum were collected after 21 days. The alanine aminotransferase (ALT), aspartate transaminase (AST), creatinine (Cr), and blood urea nitrogen (BUN) in serum were measured through assay kits. Meanwhile, H&E staining was performed on the main organs.

### Pharmacokinetics of PEG-CNPs

Healthy mice (*n* = 3) were intravenously injected with PEG-CNPs (1.5 mg/kg). At different time points post-injection (0.25 h, 0.5 h, 1 h, 4 h, 12 h, and 24 h), 15 μL of blood samples were collected from the mouse tail. The concentrations of PEG-CNPs in collected blood samples were quantified by using ICP-MS. Last, the calculation of related pharmacokinetic parameters were realized by WinNonlin 3.3 software.

### Biodistribution of PEG-CNPs

Healthy mice (*n* = 3) and colitis mice (*n* = 3) were intravenously administrated with 1.5 mg/kg PEG-CNPs. The main organs (heart, liver, spleen, lung and kidney) and colons were collected at different times (2 h, 1 day and 5 day), and the concentration of cerium in these organs were measured by ICP-MS.

### Colon-targeted analysis

To test the ability of PEG-CNPs to target inflammatory sites of the colon, healthy mice and colitis mice (*n* = 3, per group) were intravenously administered with PEG-CNPs (1.5 mg/kg). Mice were euthanized after 0.5 h and colons were excised. Quantifiable amounts of colons were incubated with nitric acid at 60 °C for 12 h. After dilution, the cerium levels were quantified by using ICP-MS.

### Ameliorative effects of PEG-CNPs on experimental colitis

In this study, mice were randomly divided into 3 groups (*n* = 10, per group): 1) Control-no colitis induced (i.v., normal saline), 2) 0.25% TNBS (i.v., normal saline) and 3) TNBS + PEG-CNPs (i.v., 1.5 mg/kg). The number of experimental animals [56] and the dose of PEG-CNPs [57, 58] were determined as previously reported, respectively. Briefly, after fasting overnight, the mice were intravenously injected with normal saline or 1.5 mg/kg PEG-CNPs 2 h after TNBS injection. The mice were sacrificed at 6 days after injection.

Bodyweights were assessed daily during the 5-d experimental period. The disease activity index (DAI) of mice was scored from day 1 according to the scoring criteria (Table S2) [59]. At the end of the experiment, the blood samples of mice were collected under isoflurane anesthesia. Then, mice were sacrificed and the entire colons were excised. The colon length of each mice was measured, and the colons were washed with precooled normal saline. Then, portions of colon samples were fixed with 4% paraformaldehyde and used for histological assessment. The remaining colon tissue samples were used for determining myeloperoxidase (MPO) activity by an assay kit. Additionally, the colon tissue samples were also used for determining malondialdehyde (MDA) content and SOD activity by assay kits following the provider’s instructions. At the end of the experiment, mice in all groups were sacrificed, and then their entire colons were fixed by incubation with 4% paraformaldehyde, and then embedded in paraffin. Tissue sections were stained with hematoxylin and eosin (H&E), and histological observations were performed by using by an Axioskop40 light microscopy (Carl Zeiss, Jena, Germany).

### In vivo intestinal permeability assay

In vivo assay of intestinal permeability was performed using FITC-dextran as described previously [60]. Briefly, mice were deprived of food and water for 4 h and then orally gavaged with 0.6 mg/g body weight of 4 kDa FITC-dextran (FD4, Sigma). Blood samples were collected after 3 h, and FITC fluorescence intensity was measured (excitation, 485 nm; emission, 520 nm). The concentrations of FITC-dextran were determined using a standard curve of serial dilution of FITC-dextran in mouse serum.

### Inflammatory cytokines determination of colon tissues and serum

To determine the concentrations of inflammatory cytokines in the colon, the colon segment in precooled normal saline was homogenized (1:10 w/v) at 4 °C. The levels of inflammatory cytokines (TNF-α, IL-1β, IL-10 and TGF-β1) in the supernatants were measured by ELISA kits (Beyotime, China).

The blood samples of mice were collected for determining the concentrations of serum inflammatory cytokines by ELISA kits (Beyotime, China).

### Immunohistochemistry assay of the colon tissues

The colon sections in paraffin were deparaffinized and immersed in 3% H_2_O_2_-PBS buffer for 15 min to eliminate the interference of endogenous peroxidase. The sections were washed for three times, and blocked in 10% bovine serum albumin containing 0.5% Triton X-100. Antibodies against HIF-1α, CD86, ZO-1, occludin-1 were implemented to mark relevant proteins, respectively. After secondary antibody incubation and substrate reaction, the final immunohistochemistry analysis was performed using Axioskop40 light microscopy (Carl Zeiss, Jena, Germany).

### Statistical analysis

Statistical analyses were performed with SPSS Version 19.0 (IBM Corporation, Armonk, New York, USA), GraphPad Prism version 8.0 (GraphPad Software) and Image J version 1.8.0. All data with normal distribution are presented as mean ± standard deviation (SD). For statistical comparison, we performed two-tailed Student’s t-test and one-way ANOVA. Statistical significance was indicated as *P* < 0.05.

## Results

### Synthesis and characterization of PEG-CNPs

**Fig. 1:**
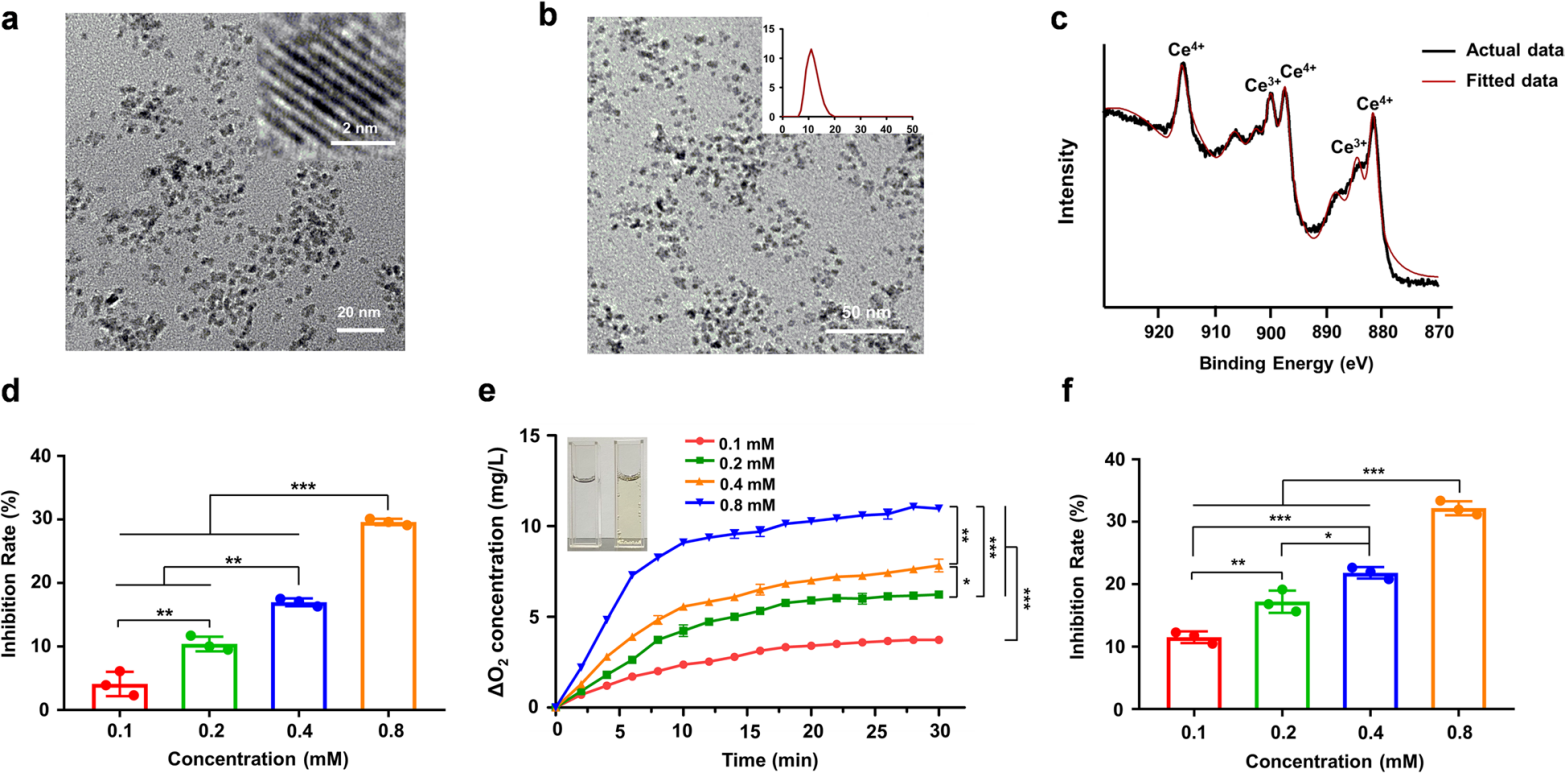
Characterization of OA-CNPs and PEG-CNPs. **a** TEM image of OA-CNPs. Inset, high-resolution TEM image of OA-CNPs. **b** TEM image and dynamic light scattering analysis (inset) of PEG-CNPs. **c** XPS spectrum of PEG-CNPs identifies the state of valence and binding energy peaks for Ce^3+^ (peaks at 884.5, 900.8 and 903.1 eV) and Ce^4+^ (peaks at 881.7, 888.5, 897.5, 906.7 and 916.2 eV). **d** The superoxide-mimicking ability of PEG-CNPs. **e** The catalase-mimicking ability of PEG-CNPs. **f** The hydroxyl radical eliminating capacity of PEG-CNPs. Data represent means ± s.d. *n* = 3. *P < 0.05, **P < 0.01, ***P < 0.001

The hydrophobic OA-CNPs with a diameter of ~ 4 nm were fabricated via a sol–gel reaction (Fig. S1). TEM images (Fig. 1a) and the XRD pattern (Fig. S2) indicated the successful synthesis of OA-CNPs. OA-CNPs were further modified with DSPE-PEG_2000_. The PEG-CNPs were dispersed in water with a hydrodynamic size of ∼11.2 nm (Fig. 1b) and a zeta potential of -19.1 ± 1.32 mV, showing good colloidal stability in media for one week (Fig. S3). The XPS analysis confirmed the Ce^3+^ and Ce^4+^ co-exist on the surface of PEG-CNPs, indicating the catalytic activities of PEG-CNPs (Fig. 1c).

Next, we evaluated the catalytic activities of PEG-CNPs including SOD-mimicking activity, CAT-mimicking activity, and hydroxyl radical eliminating capacity. PEG-CNPs demonstrated SOD-like activity (Fig. 1d), and their ability to catalyze the decomposition of superoxide radicals to H_2_O_2_ increased significantly with increasing concentration. PEG-CNPs can effectively decompose H_2_O_2_ into O_2_, indicating that PEG-CNPs have CAT-mimicking activity (Fig. 1e) and the enzymatic activity is concentration-dependent (Fig. 1e). Furthermore, as the concentration increases, PEG-CNPs exhibited enhanced ·OH eliminating activity (Fig. 1f). The results demonstrated that the PEG-CNPs have excellent ROS scavenging and O_2_ generating capabilities.

### Biocompatibility, pharmacokinetics and biodistribution studies of PEG-CNPs

First, we detected the cytotoxic effects of PEG-CNPs against HT-29 cells or RAW 264.7 cells after 24 h incubation. As shown in Fig. S4, PEG-CNPs showed no obvious cytotoxicity incubation with HT-29 cells or RAW 264.7 cells for 24 h, indicating negligible cytotoxicity of PEG-CNPs at tested concentrations (1.25–40 μM). We also conducted a hemolysis assay to evaluate PEG-CNPs hemocompatibility. The hemolysis rate demonstrated that PEG-CNPs had good hemocompatibility within 50 μM (Fig. S5). Toxic side effects of nanomaterials in vivo are an important challenge for their future biomedical applications. Therefore, we performed the histological analysis in the main organs by H&E staining in the colitis mice after PEG-CNPs treatment. As shown in Fig. S6, the sections of major organs, including heart, liver, spleen, lung, and kidney, did not exhibit the obvious inflammation or other pathological changes after treatment with PEG-CNPs for 5 days in colitis mice. Furthermore, we further examined the long-term toxicity of PEG-CNPs in healthy mice. After 21 days of intravenous injection, blood biochemistry analyses revealed that the levels of ALT and AST as well as BUN and Cr in the PEG-CNPs group were comparable to those in the control group (Fig. S7a-d). Similarly, the pathological analysis revealed that PEG-CNPs demonstrated no obvious damage to these major organs of the mice (Fig. S7e). Together, these results indicated that PEG-CNPs with good biocompatibility are promising for further biomedical applications.

Next, we performed the pharmacokinetic study of PEG-CNPs. The intracorporal pharmacokinetic course of PEG-CNPs fitted well with the classic two-compartment pharmacokinetic model, in which the terminal elimination half-lives of the central component and peripheral component were 0.41 h and 69.32 h, respectively (Fig. S8 and Table S3). We further explored the biodistribution of PEG-CNPs in healthy mice and colitis mice. As shown in Fig. S9, in healthy mice, the liver had the highest concentration of PEG-CNPs, followed by the spleen and kidney, with small distributions in the heart, lung and colon. In colitis mice, the distribution of PEG-CNPs was similar to that of healthy mice, but the Ce concentration in colonic tissues was significantly higher than that in healthy mice, indicating that PEG-CNPs can accumulate at the site of colonic inflammation through the EPR effect. The colon targeting study of PEG-CNPs also confirmed this result (Fig. S10). In addition, as in other organs, the contents of PEG-CNPs in inflamed colonic tissues gradually decreases over time; however, their elimination rate in inflamed colonic tissues was lower than in other organs, and this long-term retention property in inflamed colonic tissues favored their anti-inflammatory effect (Fig. S9). These results showed that CNPs could exert long-lasting anti-inflammatory effects by enriching at the site of colonic inflammation through EPR effect.

### Ameliorative effects of PEG-CNPs on experimental colitis

**Fig. 2:**
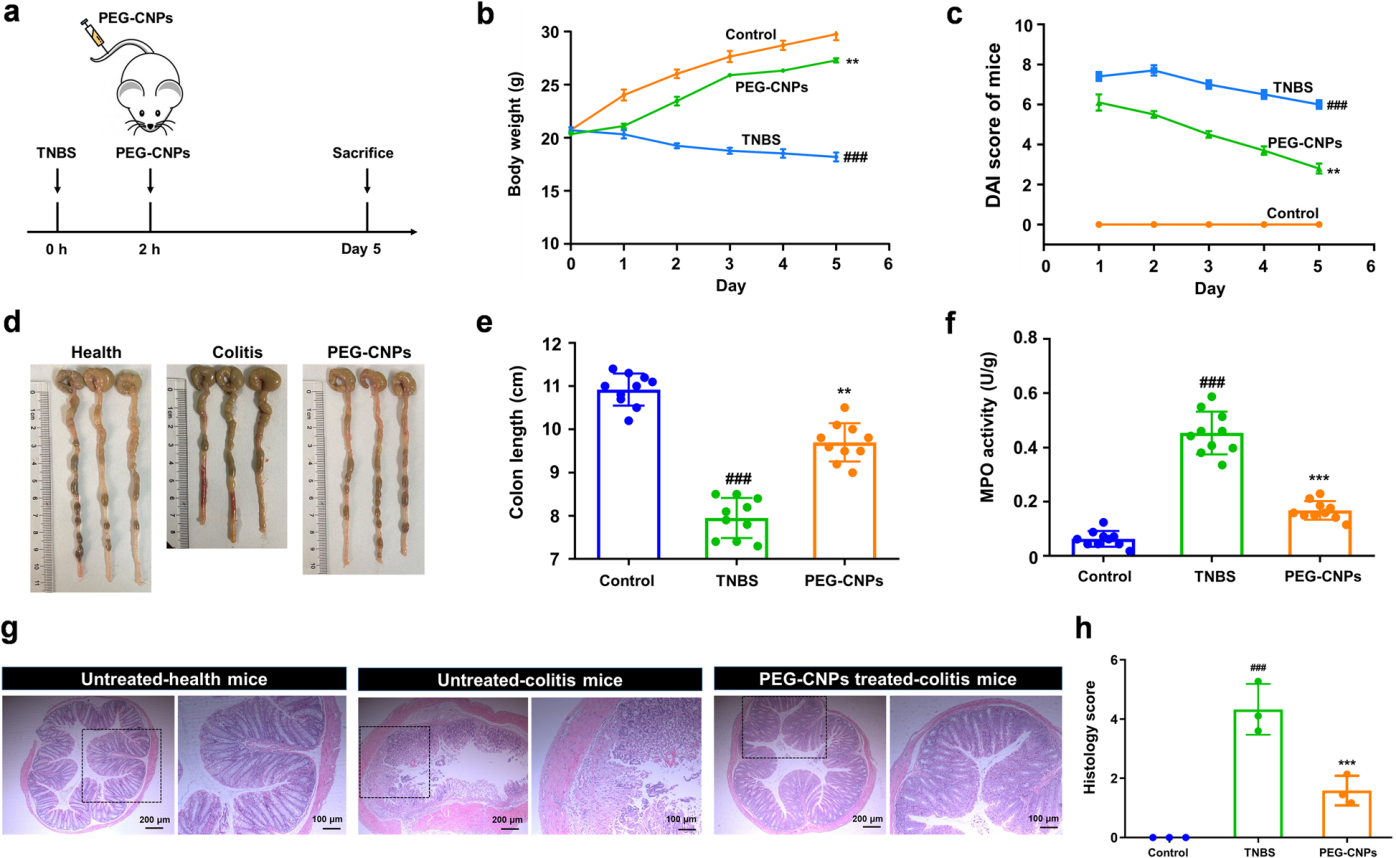
PEG-CNPs ameliorate the TNBS-induced colitis in mice. **a** Experimental scheme of PEG-CNPs treating strategy in mice. The mice were treated with normal saline (intravenous injection) or PEG-CNPs (intravenous injection, 1.5 mg/kg) at 2 h after the TNBS administration and mice were sacrificed at day 5. **b** The body weights in each group for 5 d. **c** The DAI scores in each group for 5 d. **d-h** On day 5, animals were euthanized and colon images **d**, colon length **e**, and colonic MPO activity **f** were measured. **g** Representative histopathology changes in the colon. **h** The histopathology scores in the colon (*n* = 3). Data represent means ± s.d. *n* = 10. ^###^*P* < 0.001 vs. control group; ^**^P < 0.01, ^***^P < 0.001 vs. TNBS group

Encouraged by the nanozyme-like performance of PEG-CNPs, the therapeutic efficacy in the TNBS-induced colitis mouse model was investigated. The mice were intravenously administrated with normal saline or 1.5 mg/kg PEG-CNPs at 2 h after colitis induction, and the therapeutic effect was evaluated 5 days later (Fig. 2a). This study evaluated the ameliorative effects of PEG-CNPs on TNBS-colitis using several indicators, including body weight, DAI, colon length, MPO activity and histological analysis. Mice in the TNBS group exhibited a decreasing trend in body weight, while there was a moderate increase in body weight in the PEG-CNPs group (Fig. 2b). The colon was significantly shorter in mice in the TNBS group (7.9 ± 0.4 cm) than in the control group (10.9 ± 0.4 cm) due to the colon inflammation, mucosal damage, and edema in the colon, while mice treated with PEG-CNPs had significantly improved colon lengths (9.7 ± 0.4 cm), nearly being restored to normal (Fig. 2d and e). Mice in the colitis group also exhibited a significant increase in DAI compared with those in the control group, while mice in the PEG-CNPs group had lower DAI than those in the colitis group (Fig. 2c). MPO, a glycosylase present in neutrophil and monocyte granules, can be acts as a biological marker to assess the disease status of UC [61]. Compared with the control group, MPO activity greatly increased in the colitis mice but significantly decreased in the PEG-CNPs treatment group, indicating PEG-CNPs reduced the infiltration of inflammatory cells in the colon (Fig. 2f). The histological analysis revealed that mice with colitis exhibited severe destruction of mucous membranes, massive inflammatory cell infiltration, goblet cell depletion, and disappearing crypt structures. In contrast, administration of PEG-CNPs significantly attenuated the histological signs of colitis, including tissue injury and inflammatory cell infiltration, indicating the significant protective effect of PEG-CNPs in colitis mice (Fig. 2g and h). These results indicate that PEG-CNPs can efficiently relieve the symptom of TNBS-induced colitis.

### *In vitro* hypoxia attenuating and ROS scavenging of PEG-CNPs in intestinal epithelial cells

Previous studies have found that physiological HIF-1α expression provides a barrier-protective function in intestinal disease [62, 63]. We therefore next investigated the effects of PEG-CNPs treatment on HIF-1α expression in intestinal epithelial cells.

**Fig. 3:**
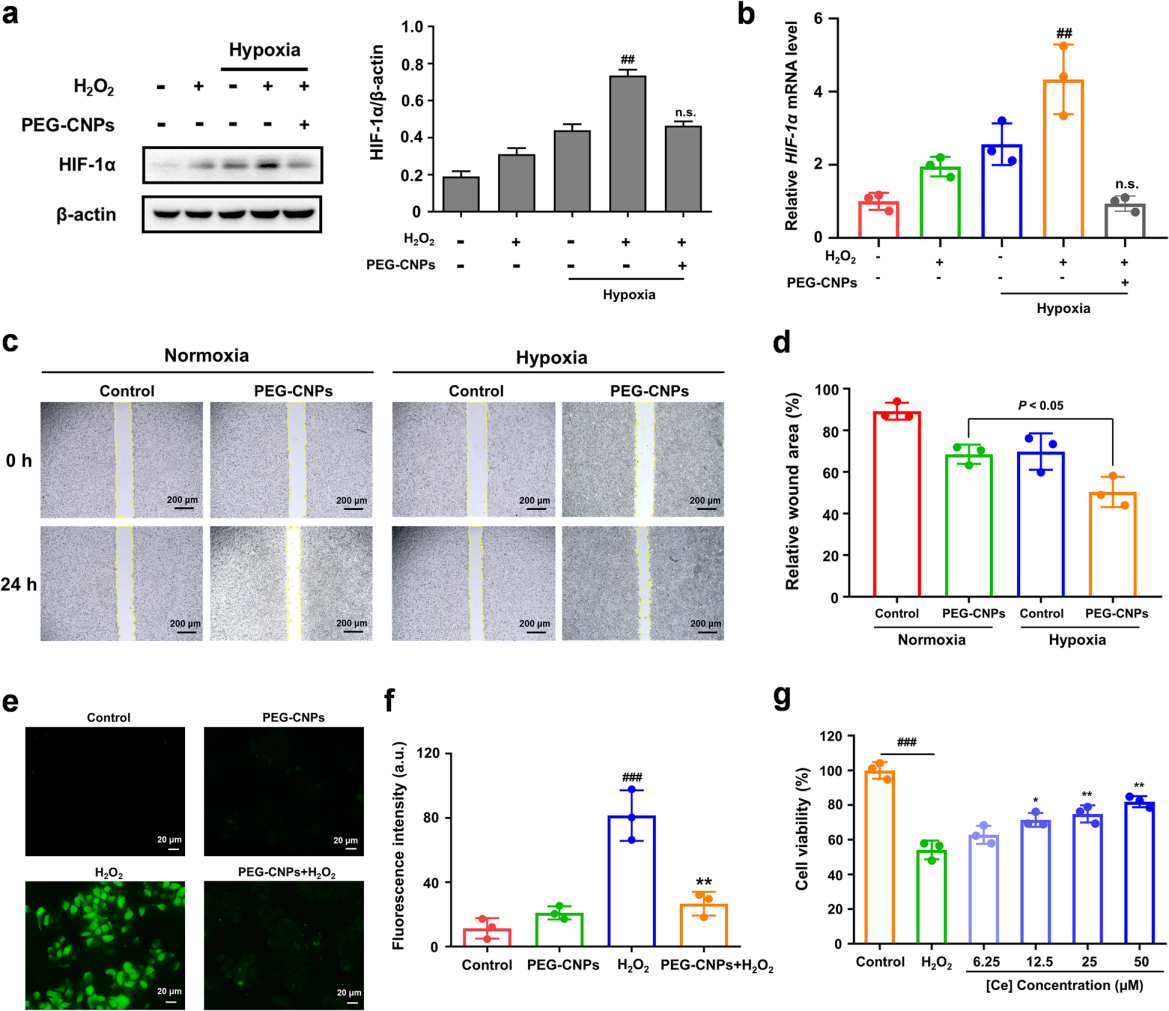
Hypoxia attenuating and ROS scavenging of PEG-CNPs in intestinal epithelial cells. **a** Western blot analysis of HIF-1α (β-actin, loading control) in HT-29 cells with different treatment strategies. **b** qRT-PCR analysis of HIF-1α in HT-29 cells with different treatment strategies. **c** Migration ability of HT-29 cells with PEG-CNPs treatment. **d** Relative wound area with PEG-CNPs treatment. **e** Representative ROS staining of HT-29 cells upon different treatments using DCFH-DA probe. **f** Quantification of fluorescence of DCFH-DA probe (staining ROS) in HT-29 cells upon different treatments. **g** Viabilities of HT-29 cells treated with PEG-CNPs and H_2_O_2_ show that PEG-CNPs protect cells from oxidative stress-induced cell death. Data represent means ± s.d. *n = *3. ^#^*P* < 0.05, ^##^*P* < 0.01, ^###^*P* < 0.001 vs. control group; ^*^P < 0.05, ^**^P < 0.01, ^***^P < 0.001 vs. H_2_O_2_ group; no significance (n.s.) vs. normoxia group

After 24 h of incubation using FITC-PEG-CNPs, cellular CNP uptake in HT-29 cells was confirmed using fluorescence microscopy (Fig. S11a). We verified the hypoxia-attenuating ability of PEG-CNPs by evaluating intracellular HIF-1α expression levels. HT-29 cells were incubated under hypoxia atmosphere (1% O_2_, 5% CO_2_, and 94% N_2_) and H_2_O_2_ for 4 h with PEG-CNPs treatment. As shown in Fig. 3a and b, we found that the initial HIF-1α expression in intestinal epithelial cells was comparable to that for “physiological hypoxia” under hypoxia condition, indicating that this condition could simulate the physiological hypoxia of intestinal epithelium. After adding H_2_O_2_, HIF-1α levels in HT-29 cells under hypoxia significantly increased. In contrast, HIF-1α levels significantly decreased to the initial level under hypoxia with PEG-CNPs treatment, which might be related to the O_2_ generation of PEG-CNPs during ROS scavenging.

Previous studies have found that stable HIF-1α expression in intestinal epithelial cells is helpful for repairing the intestinal mucosal barrier. Based on the hypoxia- attenuating ability of PEG-CNPs, we then explored the migration ability of HT-29 cells with PEG-CNPs treatment using a cell scratch test. As shown in Fig. 3c, HT-29 cells were cultured in a 12-well plate and scratched using a pipette tip to create scratches. After 24 h of incubation, marked migration of HT-29 cells was observed in the scratched area after PEG-CNPs treatment. The wound areas in the PEG-CNPs group under hypoxia were significantly smaller than those in the other groups (Fig. 3c and d).

Considering the ROS scavenging capability of PEG-CNPs (Fig. 1d-f), we further evaluated the intracellular ROS levels in H_2_O_2_ treated HT-29 cells using a 2’,7’-dichlorodihydrofluorescein diacetate (DCFH-DA) assay. As expected, a significant increase in ROS level was detected after H_2_O_2_ stimulation, which was markedly eradicated by the PEG-CNPs (Fig. 3e and f). To further confirm the anti-oxidative effect of PEG-CNPs in vitro, the cytoprotective effect of PEG-CNPs against H_2_O_2_ in HT-29 cells was determined. As illustrated in Fig. 3g, PEG-CNPs significantly decreased the H_2_O_2_-induced cell damage in a dose-dependent manner within 50 μM.

**Fig. 4:**
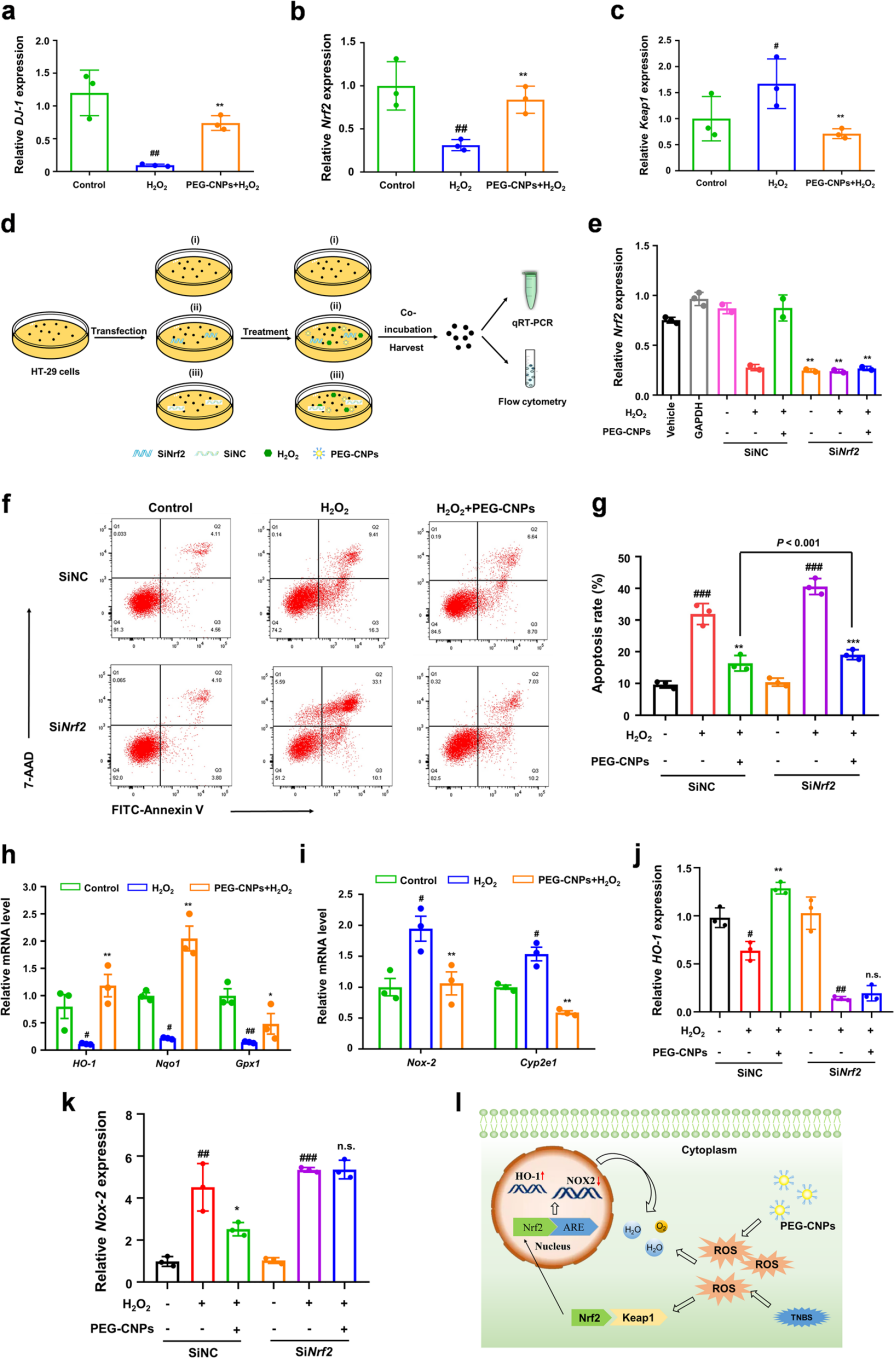
PEG-CNPs counteract oxidative stress via activating *Nrf2/Keap1* signaling pathway. Relative mRNA expressions of *DJ-1* **a**, *Nrf2* **b**, and *Keap1* **c** in HT-29 cells after different treatments. **d** Schematic diagram of the experimental protocol to evaluate the role of *Nrf2* by small interfering RNA (SiRNA) in vitro using qRT-PCR analysis and flow cytometry. **e** Relative mRNA expressions of *Nrf2* in Si*Nrf2*-transfected HT-29 cells. **f** Flow cytometry analysis of apoptosis in Si*Nrf2*-transfected HT-29 cells. **g** Apoptosis rate of HT-29 cells. **h** The anti-oxidative genes (*Nqo1*, *Gpx1*, *HO-1*) and **i** pro-oxidative genes (*Nox-2*, *Cyp2e1*) expression levels in HT-29 cells treated with PEG-CNPs and H_2_O_2_. Relative mRNA expressions of *HO-1* **j** and *Nox-2* **k**. **l** Schematic representation of the mechanism of PEG-CNPs in reducing ROS level. PEG-CNPs decompose DDP-induced H_2_O_2_ into H_2_O and O_2_. Meanwhile, the minimally residual ROS activate the *Nrf2/Keap1* signaling pathway. Specifically, Nrf2 moves into the nucleus and subsequently binds to the antioxidant response elements (ARE), leading to the upregulation of the antioxidant gene (*HO-1*) and downregulation of the pro-oxidant gene (*Nox-2*). These gene regulations can further detoxify ROS. Data represent means ± s.d. *n = *3. ^#^*P* < 0.1, ^##^*P* < 0.01, ^###^*P* < 0.001 vs. control group; ^*^P < 0.1, ^**^P < 0.01, ^***^P < 0.001 vs. H_2_O_2_ group

*Nrf2* is known to be a major regulator of the cellular antioxidant pathways, which would be decreased due to excessive ROS. In response to oxidative stress, *Nrf2* can dissociate from *Keap1* and translocate into the nucleus, which plays a key role in initiating antioxidant response by activating a network of genes (such as *Gpx1*, *Nqo1* and *HO-1*) [64, 65]. In this regard, we performed qRT-PCR analysis to determine the levels of *Nrf2*, *DJ-1* and *Keap1*. The results indicated that the level of *DJ-1* inhibited by oxidative stress could be reversed by PEG-CNPs (Fig. 4a). Meanwhile, PEG-CNPs upregulated *Nrf-2* while downregulated *Keap1*, which contributed to the anti-oxidative ability of PEG-CNPs (Fig. 4b and c). To further investigate the role of *Nrf2* in PEG-CNPs-mediated antioxidant activity, we examined whether *Nrf2* silence could offset the protective effects of PEG-CNPs on H_2_O_2_-induced cytotoxicity (Fig. 4d and e). Studies have shown that the apoptosis of colonic epithelial cells can lead to the destruction of mucosal barrier and increase of inflammatory cells infiltration, and further aggravate mucosal injury [66]. Interestingly, PEG-CNPs would fail to prevent the HT-29 cells apoptosis after *Nrf2* silence, suggesting that *Nrf2* is a crucial factor in inhibiting apoptosis of PEG-CNPs (Fig. 4f and g). Furthermore, the status of DJ-1/Nrf2/Keap1 axis was analyzed by qRT-PCR analysis. Anti-oxidative stress genes (*HO-1, Nqo1*, *Gpx1*) up-regulation and pro-oxidative stress genes (*Nox-2*, *Cyp2e1*) in HT-29 cells treated with PEG-CNPs (Fig. 4h and i). In contrast, the relative levels of anti-oxidant genes and pro-oxidant genes in HT-29 cells with *Nrf2* knockdown were no longer sensitive to PEG-CNPs treatment (Fig. 4j and k). These results indicated that PEG-CNPs were involved in activating *Nrf2* to scavenge ROS (Fig. 4l), thereby preventing colonic epithelial cell apoptosis. In summary, the antioxidative activity of PEG-CNPs should be mediated by directly scavenging ROS and the reconstitution of the Nrf2-mediated cellular anti-oxidative system.

### *In vitro* hypoxia attenuating, ROS scavenging and inflammatory regulation of macrophages induced by PEG-CNPs

**Fig. 5:**
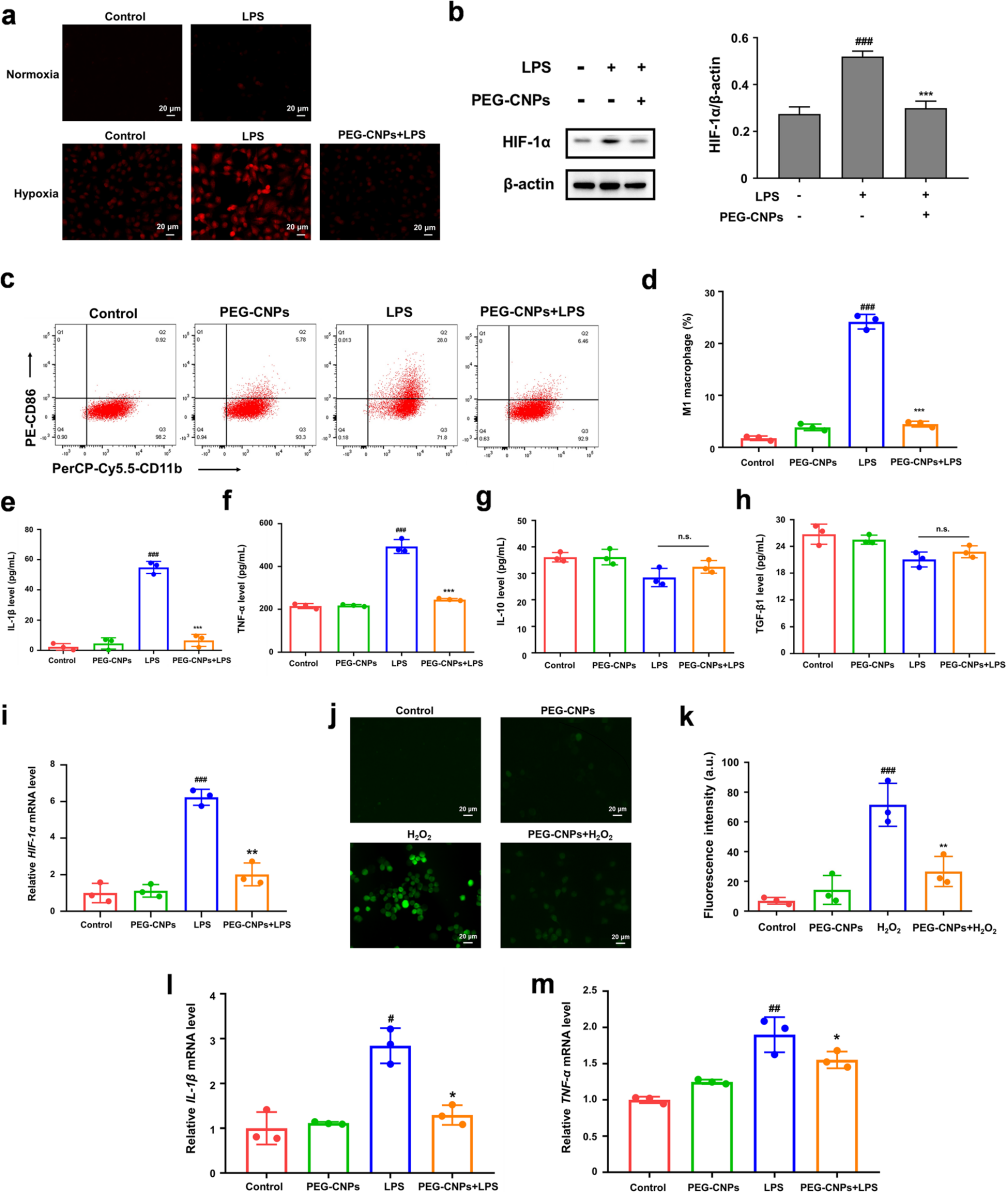
Hypoxia attenuating and ROS scavenging of macrophages induced by PEG-CNPs in RAW 264.7 cells. **a** Fluorescence images of RAW 264.7 cells after different treatments. The hypoxia level was indicated by intracellular oxygen indicator [Ru(dpp)_3_]Cl_2_. Cells in hypoxia (1% O_2_) model were used as the control group. Cells in normoxia (21% O_2_) was used as the positive control. **b** Western blot analysis of HIF-1α (β-actin, loading control) in RAW 264.7 cells with different treatment strategies. **c** Flow cytometry analysis of CD86^+^ macrophages with LPS simulation. **d** Quantification of the percentage of CD86^+^ macrophages. IL-1β **e**, TNF-α **f**, IL-10 **g** and TGF-β1 **h** levels in RAW 264.7 cells with different treatment strategies. **i** qRT-PCR analysis of *HIF-α* in RAW 264.7 cells. **j** Representative ROS staining of RAW 264.7 cells upon different treatments using DCFH-DA probe. **k** Quantification of fluorescence of DCFH-DA probe (staining ROS) in RAW 264.7 cells upon different treatments. qRT-PCR analysis of *IL-1β* **l** and *TNF-α* **m** in RAW 264.7 cells. Data represent means ± s.d. *n = *3. ^#^*P* < 0.05, ^##^*P* < 0.01, ^###^*P* < 0.001 vs. control group; ^*^P < 0.05, ^**^P < 0.01, ^***^P < 0.001 vs. LPS group

Hypoxia of macrophages and the overproduction of ROS at the inflammation site of UC can aggravate the colon inflammation and induce the M1 polarization of macrophages [67]. Ameliorating hypoxia of macrophages and decreasing ROS levels can therefore attenuate pro-inflammatory macrophages, and effectively improve UC. To verify the hypoxia-attenuating ability of PEG-CNPs, we evaluated HIF-1α levels in vitro and intracellular oxygen levels. After 24 h of incubation with FITC-PEG-CNPs, fluorescence images illustrated the efficient cellular uptake of PEG-CNPs by RAW 264.7 cells (Fig. S11b). We also found up-regulation of HIF-1α expression in RAW 264.7 cells incubated with LPS for 24 h, which was consistent with previous studies [68]. We therefore carried out subsequent in vitro hypoxia-condition treatment with only LPS. RAW 264.7 cells were then incubated with LPS, and evaluated using [Ru(dpp)_3_]Cl_2_. [Ru(dpp)_3_]Cl_2_ is a typical indicator of intracellular oxygen which shows distinct red luminescence at 613 nm under hypoxia while the red luminescence is quenchable by oxygen [69]. As illustrated in Fig. 5a, compared with the LPS group, the treatment with PEG-CNPs led to significant decreases in red luminescence, indicating elevation of the intracellular O_2_ level. Western blotting indicated that HIF-1α expression was significantly increased after LPS stimulation, which was attenuated by PEG-CNPs treatment (Fig. 5b).

Many macrophages are recruited and accumulated in the ulcerative colon, causing persistent inflammation. M1 macrophages produce pro-inflammatory cytokines and ROS [48]. To test the effect of PEG-CNPs on the inflammatory response in UC, the proportion of CD86^+^ cell was measured by flow cytometry. Interestingly, the proportion of CD86^+^ cells increased significantly due to the LPS stimulation, which could be reversed by PEG-CNPs treatment (Fig. 5c and d) and the morphological analysis of RAW 264.7 cells (Fig. S12). Pro-inflammatory M1 markers expression, including IL-1β and TNF-α, were significantly increased after LPS stimulation, which could be reversed by PEG-CNPs treatment (Fig. 5e and f). Moreover, PEG-CNPs were found to have increased levels of anti-inflammatory cytokines (IL-10 and TGF-β1) compared to the LPS group, but there was no statistically significant difference (Fig. 5g and h). Notably, treatment with PEG-CNPs markedly reduced the HIF-1α expression levels (Fig. 5i). A significant increase in the ROS level of RAW 264.7 cells was also detected after H_2_O_2_ stimulation, which was markedly attenuated by PEG-CNPs (Fig. 5j and k). HIF-1α, ROS, and M1 marker levels showed similar tendencies after the PEG-CNPs intervention, indicating that reducing HIF-1α expression and scavenging ROS play crucial roles in the suppression of M1 macrophages activation [55, 70]. Similarly, qRT-PCR analysis of RAW 264.7 cells showed that PEG-CNPs treatment significantly reduced the mRNA expression level of M1 markers including *IL-1β* and *TNF-α* (Fig. 5l and m). Together these observations indicated that PEG-CNPs can attenuate hypoxia of macrophages and scavenge excess ROS in macrophages via generate O_2_ and scavenging ROS, and the successful inhibition of M1 macrophages activation.

### Restoration of colonic epithelium and inhibition of M1 macrophages activation induced by PEG-CNPs in the colitis mice

**Fig. 6:**
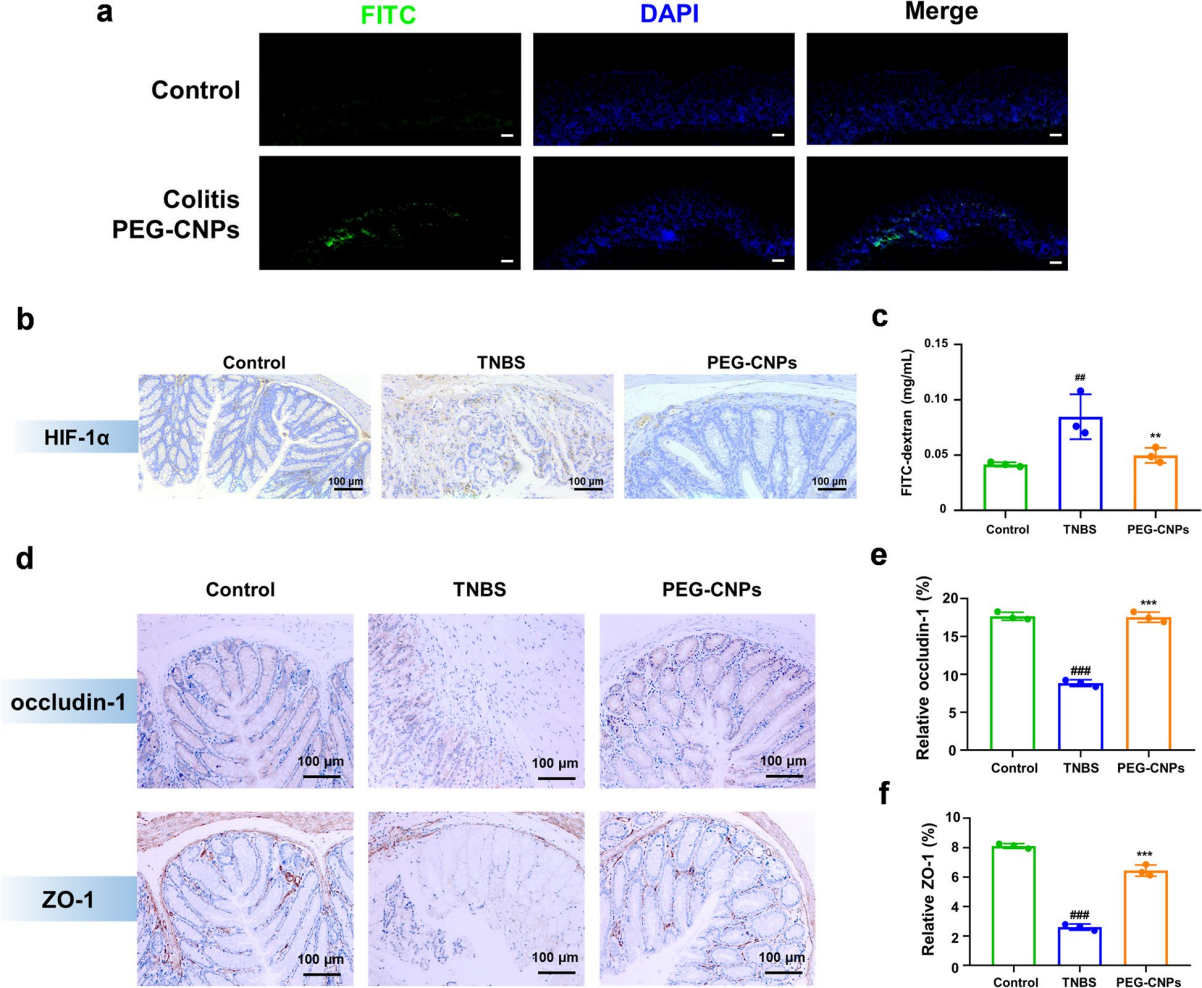
In vivo restoration of colonic epithelium by PEG-CNPs. **a** CLSM images to track the intracellular fate of FITC-PEG-CNPs after injection (Scale bars: 100 μm). **b** Immunohistochemical analysis of HIF-1α of colon tissues from healthy or TNBS-colitis mice. **c** Intestinal barrier functions were measured the FITC-dextran signal in blood. **d** Immunohistochemical analysis of occludin-1 and ZO-1 of colon tissues from healthy or TNBS-colitis mice. Statistical analysis of occludin-1 **e** and ZO-1 **f** in immunohistochemical staining. Data represent means ± s.d. *n = *3. ^##^*P* < 0.01, ^###^*P* < 0.001 vs. control group; ^**^P < 0.01, ^***^P < 0.001 vs. TNBS group

To further elucidate the mechanism of the protective effect of UC, we investigated the impact of PEG-CNPs on colonic epithelium restoration and colonic M1 macrophage in colitis mice. We first isolated colon tissues to track the intracellular fate of FITC-PEG-CNPs at 0.5 h after the injection. Confocal laser scanning microscopy images of colon tissues illustrated that FITC-PEG-CNPs were distributed more in the inflammatory cell infiltration layer of colon tissues than in the colonic epithelium (Fig. 6a). We then determined whether O_2_ generated by PEG-CNPs alleviated hypoxia in inflamed colons. Immunohistochemical HIF-1α staining demonstrated that PEG-CNPs administration prominently inhibited HIF-1α expression in the inflammatory cell infiltration layer of colon tissues (Fig. 6b).

We investigated the effect of PEG-CNPs on colonic epithelium with disrupted intestinal barrier functions caused by TNBS. The effects of HIF-1α protein stabilization on barrier protection are thought to be attributable to tight-junction (TJ) protein (such as claudin-1) regulation [71, 72]. In the present study, colitis mice administered with PEG-CNPs had normalized ZO-1 and occludin-1 levels, which are tight-junction proteins that play vital roles in gut homeostasis (Fig. 6d-f) [3]. Furthermore, compared with the TNBS group, PEG-CNPs prevented systemic FITC-dextran exposure in colitis mice (Fig. 6c), demonstrating intestinal barrier function restoration. Collectively these results indicated that PEG-CNPs could enhance HIF-1α protein stability in intestinal epithelial cells and restore intestinal barrier functions by increasing the expression of tight-junction proteins [73].

**Fig. 7:**
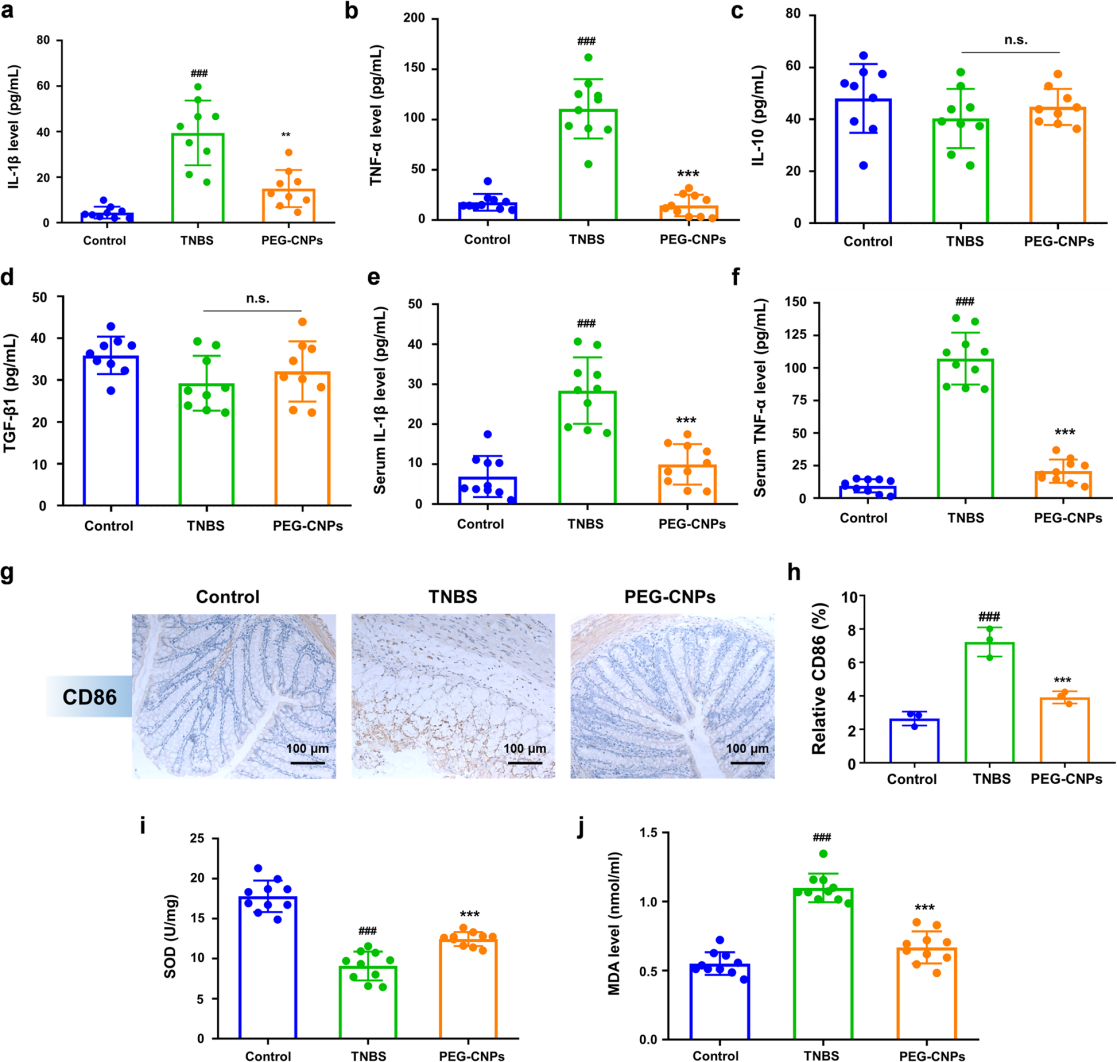
In vivo inhibition of M1 macrophages activation induced by PEG-CNPs. Pro-inflammatory cytokines **a** IL-1β and **b** TNF-α levels and anti-inflammatory cytokines **c** IL-10 and **d** TGF-β1 levels with PEG-CNPs treatment in the colon tissues (*n = *10). Pro-inflammatory cytokines **e** IL-1β and **f** TNF-α levels with PEG-CNPs treatment in the blood serum (*n = *10). **g** Immunohistochemical analysis of CD86 of colon tissues from healthy or TNBS-colitis mice (*n = *3). **h** Statistical analysis of CD86 in immunohistochemical staining (*n = *3). The SOD activity **i** and the MDA level **j** with PEG-CNPs (*n* = 10). Data represent means ± s.d. ^###^*P* < 0.001 vs. control group; ^**^P < 0.01, ^***^P < 0.001 vs. TNBS group

The role of PEG-CNPs in suppressing the pro-inflammatory macrophage activation in vivo, the levels of CD86^+^ cells in the colon were measured immunohistochemically. As shown in Fig. 7g and h, the CD86 level was increased after inducing colitis, whereas PEG-CNPs injection prominently reduced that level. Besides, consistent with the in vitro results (Fig. 5e and f), pro-inflammatory cytokine levels (including IL-1β and TNF-α) in the colon tissues and blood serum were lower in the PEG-CNPs group than in the colitis group (Fig. 7a, b, e and f). Moreover, PEG-CNPs were found to have increased levels of anti-inflammatory cytokines (IL-10 and TGF-β1) compared to the TNBS group, but there was no statistically significant difference (Fig. 7c and d). It implied to us that PEG-CNPs exerts its anti-inflammatory effects on colitis by primarily suppressing the pro-inflammatory macrophage activation. To further examine the anti-oxidative effect of PEG-CNPs in vivo, we measured MDA levels and SOD activity in colon tissues. The MDA level was elevated and SOD activity was inhibited after TNBS treatment, while treatment with PEG-CNPs could reverse the imbalance of these ROS-related indicators (Fig. 7i and j). Consequently, PEG-CNPs suppress pro-inflammatory macrophage activation by alleviating hypoxia and scavenging ROS of colon inflammatory tissues, while enhancing the body’s ability of ROS scavenging.

## Discussion

UC is a chronic inflammatory bowel disease, and is accompanied by intestinal inflammation and mucosal tissue damage. ROS overproduction and excessive hypoxia play pivotal roles in the initiation and progression of UC. Due to the great ROS scavenging effect of CNPs, some studies have prepared oral drug delivery systems loaded with CNPs for colitis treatment [74–77]. Although convenient for oral administration, CNPs based on this mode of administration mostly exert their corresponding therapeutic effects by scavenging ROS from the surface of inflamed tissues and intestinal lumen, but cannot act deep into inflamed tissues and inflammatory cells, and the preparation of drug delivery systems is complicated. Therefore, another study has reported PEGylated hollow cerium oxide nanoparticles for intravenous administration in the treatment of colitis [78]. However, the synthesis of hollow cerium oxide nanoparticles is complex. The synthesis process of PEG-CNPs synthesized in this study is much simpler; and the intravenous administration facilitates the penetration of cerium oxide nanoparticles into colonic inflammatory tissues and exerts anti-inflammatory effects. Notably, the administration dose of PEG-CNPs (1.5 mg/kg) was much lower than that of above mentioned hollow cerium oxide nanoparticles (20 mg/kg) used for the treatment of colitis, which contributes to the safety of the treatment. The good biocompatibility, biodistribution and targeting to the inflamed colon tissue of PEG-CNPs, are important prerequisites for the treatment of UC. Hemolysis assays demonstrated the safety of intravenous administration of PEG-CNPs. In vivo safety experiments showed that therapeutic doses of PEG-CNPs (1.5 mg/kg) were not significantly toxic to the major organs of colitis mice. Besides, there was no long-term toxicity (21 days) of PEG-CNPs in healthy mice, which indicated the great biocompatibility of PEG-CNPs. In vivo distribution experiments of PEG-CNPs showed that after intravenous administration, PEG-CNPs were mainly distributed in tissues with rich blood supply, such as liver. Ce concentrations were significantly higher in the colon tissue of colitis mice than that of healthy mice, indicating that PEG-CNPs could accumulate at the site of colonic inflammation through the EPR effect. The above results were also confirmed in colon targeting experiments. In addition, the elimination rate of PEG-CNPs in colonic inflammatory tissues over time was lower than that in other organs, and this prolonged retention property in colonic inflammatory tissues facilitated their anti-inflammatory effects. Together, the results of in vivo distribution and colon tissue targeting experiments suggested that PEG-CNPs could be enriched in inflamed colon tissues through the EPR effect and enhance their therapeutic effects.

Oxidative stress plays a crucial role in mucosal damage and intestinal inflammation in UC. Therefore, therapeutic strategies that target oxidative stress signaling and ROS scavenging are promising for UC treatment. In the current study, PEG-CNPs showed SOD-mimic and CAT-mimic enzyme activities, which could decompose excessive O_2_^•−^ into H_2_O_2_, and decompose H_2_O_2_ into O_2_ in the inflammatory site, respectively (Fig. 1d-f). In the H_2_O_2_-treated HT-29 cells, PEG-CNPs could scavenge the intracellular ROS (Fig. 3e-g). Furthermore, PEG-CNPs treatment inhibited the oxidative damage in the colon induced by TNBS, including decrease in MDA and increase in anti-oxidative enzyme (SOD) activity (Fig. 7g-h). Besides, PEG-CNPs have also been used in other inflammatory diseases, such as acute kidney injury [58], drug-induced liver injury [57] and rheumatoid arthritis [46], which have pathological features similar to those of colitis (overproduction of ROS and hypoxia at the site of inflammation). This suggests that PEG-CNPs display enzymatic activity not only in the cytosol of macrophage or inflammatory microenvironment of the hypoxic colon tissue, but also in other cells (HK-2, L02 and HepG2 cells) and organs (kidney, liver and joint). In summary, the wide application of PEG-CNPs in colitis and other diseases corroborates its stable and sustainable enzymatic activity in vivo.

To further discover the molecular mechanisms that might underlie the antioxidant effect of PEG-CNPs, Nrf2/Keap1 signaling pathway were measured. Nrf2/Keap1 signaling pathway is an important defense mechanism in oxidative stress [79]. It has reported that Nrf2/Keap1 signaling pathway is participated in the UC development. In UC mice model, DSS treatment inhibit Nrf2 levels and increased Keap1 levels, indicating Nrf2/Keap1 pathway is blocked [80]. Interestingly, in the present research, the Nrf2/Keap1 signaling pathway was activated by PEG-CNPs treatment. Moreover, the inhibited expressions of Nrf2-targeted molecules (HO-1 and Nqo1) were significantly reversed after treatment with PEG-CNPs (Fig. 4). Several studies have also reported the role of PEG-CNPs in alleviating oxidative stress in other organs, such as the kidney and liver. Specifically, chemotherapy in cancer patients can cause acute kidney injury, which is correlated with the collateral damage to renal cells caused by ROS. When PEG-CNPs treat acute kidney injury, in the renal cortex, PEG-CNPs exhibit great ROS scavenging activity to break down H_2_O_2_ and subsequently regulate genes related to ROS by activating the Nrf2/Keap1 signaling pathway [58]. Besides, in drug-induced liver injury, PEG-CNPs can inhibit the pro-inflammatory macrophages by activating the Nrf2/Keap1 signaling pathway to relieve inflammation for a promoted hepatocyte regeneration of drug-induced liver injury [57]. In summary, the anti-oxidative activity of PEG-CNPs should be mediated by directly scavenging ROS and the reconstitution of the Nrf2-mediated cellular anti-oxidative system.

When the inflammation of intestinal mucosa is uncontrolled and highly activated, many inflammatory cells infiltrated the UC inflammation site, in which pro-inflammatory macrophages were dominant [81]. The high HIF-1⍺ expression level and excessive ROS generation in colonic macrophages can aggravate the intestinal inflammatory response and further induce pro-inflammatory macrophage polarization. Our study found that PEG-CNPs can passively target colonic macrophages through EPR effect, thereby decreasing HIF-1⍺, IL-1β, and TNF-α levels at the sites of inflammation (Figs. 5 and 7). Besides, in rheumatoid arthritis, poor oxygen supply to immune cells infiltrating the synovial membrane upregulates HIF-1α expression and induces ROS overproduction. Jonghoon Kim et al. developed manganese ferrite and ceria nanoparticle-anchored mesoporous silica nanoparticles that can synergistically scavenge ROS and produce O_2_ for reducing M1 macrophage levels and inducing M2 macrophages for rheumatoid arthritis treatment [46]. In conclusion, PEG-CNPs can exert anti-inflammatory effects by scavenging ROS and alleviating hypoxia to inhibit the activation of pro-inflammatory macrophages. Although PEG-CNPs can act quickly via intravenous administration, and this mode of administration is beneficial for the treatment of severe or fulminant colitis, compliance with intravenous administration is poor for the treatment of mild and moderate colitis, and it is more convenient to use oral administration. Therefore, we will further explore the use of oral colon-targeted drug delivery system to deliver PEG-CNPs in the follow-up study in order to expand the application of PEG-CNPs in the maintenance treatment of colitis.

## Conclusions

In summary, we developed PEG-CNPs with multi-enzymatic activities that can synergistically scavenge ROS and generate O_2_ to treat UC through restoring the dysregulated intestinal barrier and inhibiting the pro-inflammatory macrophages activation. It was found that PEG-CNPs had good biocompatibility. After intravenous injection of PEG-CNPs in colitis mice, they could be enriched in the site of colonic inflammation by EPR effects and scavenge ROS in impaired colon tissues, reduce HIF-1α expression in colonic macrophages by generating O_2_, reduce expression levels of pro-inflammatory cytokines and suppress the pro-inflammatory macrophages activation. Meanwhile, PEG-CNPs were found to reduce HIF-1α expression in intestinal epithelial cells by generating O_2_ based on CAT-mimicking activity, thus further upregulating TJ proteins and promoting disrupted intestinal mucosal barrier restoration. Moreover, the significant upregulation of antioxidative stress genes (Nqo1, Gpx1, and HO-1) and downregulation of pro-oxidative stress genes (Nox2 and Cyp2e1) testified that PEG-CNPs could scavenge ROS and enhance antioxidant activity via activating the Nrf2/Keap1 signaling pathway. Our study not only helps to explain the therapeutic mechanism of PEG-CNPs for UC treatment, but also provides evidence for the potential effects of PEG-CNPs in hypoxia alleviation and inhibition of pro-inflammatory macrophages activation in other hypoxia-associated inflammatory diseases, such as myocardial infarction or rheumatoid arthritis.

## Supplementary Information

**Additional file1: Fig. S1** Scheme of PEG-CNPs preparation via a modified reverse micelle method. (a) Synthetic schematic for PEG-CNPs preparation. (b) Detailed process for PEG-CNPs preparation. **Fig. S2** The XRD pattern shows the structure of OA-CNPs (a) and PEG-CNPs (b). **Fig. S3** The colloidal stability of PEG-CNPs in PBS or DMEM containing 10% fetal bovine serum (FBS) within 7 days. Data represent means ± s.d. *n* = 3. **Fig. S4** Cytotoxicity of PEG-CNPs in (a) RAW 264.7 cells and (b) HT-29 cells. Cell viability was evaluated at 24 h of exposure to PEG-CNPs. Data represent means ± s.d. *n* = 6. **Fig. S5** Photographs of red blood cells and the hemolytic activity of PEG-CNPs. Data represent means ± s.d. *n* = 3. **Fig. S6** Representative H&E staining of major organs from colitis mice at day 5 after 1.5 mg/kg PEG-CNPs treatment. **Fig. S7** Long-term toxicity of PEG-CNPs in healthy mice. Blood biochemistry analyses of ALT (a), AST (b), BUN (c) and creatinine (d) of mice at day 21 after 1.5 mg/kg PEG-CNPs treatment. (e) Representative H&E staining of major organs from mice. Data represent means ± s.d. *n* = 6. **Fig. S8 **In vivo pharmacokinetic curves of PEG-CNPs (1.5 mg/kg). Data represent means ± s.d. *n* = 3. **Fig. S9** Biodistribution of Ce element at different time points after intravenous injection of PEG-CNPs (1.5 mg/kg) in healthy mice (a) and colitis mice (b). Data represent means ± s.d. *n* = 3. **Fig. S10** Colon targeting of PEG-CNPs (1.5 mg/kg) in colon tissues. Data represent means ± s.d. *n* = 3. ^***^P < 0.001 vs. control group. **Fig. S11** Representative fluorescence images of HT-29 cells (a) and RAW 264.7 cells (b) after incubation with FITC-PEG-CNPs for 24 h. Scale bars: 50 μm. **Fig. S12** Morphology of RAW 264.7 cells after treated with LPS (-/+) (100 ng/ml) and PEG-CNPs. **Table S1** Sequences of the primers used for qRT-PCR. **Table S2** The evaluation criteria of disease activity index (DAI) of mice. **Table S3** Pharmacokinetic parameters of PEG-CNPs. Data represent means ± s.d. *n* = 3.

## Data Availability

Most of the datasets supporting the conclusions of this study are included within the manuscript and the additional files. The datasets used or analyzed during the study are available on reasonable request.

## Abbreviations

CAT: Catalase
PEG-CNPs: DSPE-PEG modified ceria nanozymes
DAI: Disease activity index
DCFH-DA: 2’,7’-Dichlorodihydrofluorescein diacetate
DSPE-mPEG_2000_: 1,2-Distearoyl-sn-glycero-3-phosphoethanolamine-N-[methoxy (polyeth-ylene glycol)-2000]
ELISA: Enzyme-linked immunosorbent assay
FITC: Fluorescein5(6)-isothiocyanate
H_2_O_2_: Hydrogen peroxide
HIF-1α: Hypoxia-inducible factor-1α
HRTEM: High resolution transmission electron microscopy
H&E: Hematoxylin and eosin
ICP-MS: Inductively coupled plasma-mass spectrometry
LPS: Lipopolysaccharide
MDA: Malondialdehyde
MPO: Myeloperoxidase
qRT-PCR: Quantitative real-time polymerase chain reaction
ROS: Reactive oxygen species
SD: Standard deviation
SOD: Superoxide
TEM: Transmission electron microscopy
TJ: Tight junction
TNBS: 2,4,6-Trinitrobenzenesulfonic acid
UC: Ulcerative colitis
XRD: X-ray powder diffraction
XPS: X-ray photoelectron spectra
