# Supplementary material for: Restoration of dysregulated intestinal barrier and inflammatory regulation through synergistically ameliorating hypoxia and scavenging reactive oxygen species using ceria nanozymes in ulcerative colitis
Source: Biomater Res. 2023 Jul 28;27:75. doi: 10.1186/s40824-023-00412-8 (PMC10375752; doi:10.1186/s40824-023-00412-8)
Supplement: Supplementary file 1 — Additional file1: Fig. S1 Scheme of PEG-CNPs preparation via a modified reverse micelle method. (a) Synthetic schematic for PEG-CNPs preparation. (b) Detailed process for PEG-CNPs preparation. Fig. S2 The XRD pattern shows the structure of OA-CNPs (a) and PEG-CNPs (b). Fig. S3 The colloidal stability of PEG-CNPs in PBS or DMEM containing 10% fetal bovine serum (FBS) within 7 days. Data represent means ± s.d. n = 3. Fig. S4 Cytotoxicity of PEG-CNPs in (a) RAW 264.7 cells and (b) HT-29 cells. Cell viability was evaluated at 24 h of exposure to PEG-CNPs. Data represent means ± s.d. n = 6. Fig. S5 Photographs of red blood cells and the hemolytic activity of PEG-CNPs. Data represent means ± s.d. n = 3. Fig. S6 Representative H&E staining of major organs from colitis mice at day 5 after 1.5 mg/kg PEG-CNPs treatment. Fig. S7 Long-term toxicity of PEG-CNPs in healthy mice. Blood biochemistry analyses of ALT (a), AST (b), BUN (c) and creatinine (d) of mice at day 21 after 1.5 mg/kg PEG-CNPs treatment. (e) Representative H&E staining of major organs from mice. Data represent means ± s.d. n = 6. Fig. S8 In vivo pharmacokinetic curves of PEG-CNPs (1.5 mg/kg). Data represent means ± s.d. n = 3. Fig. S9 Biodistribution of Ce element at different time points after intravenous injection of PEG-CNPs (1.5 mg/kg) in healthy mice (a) and colitis mice (b). Data represent means ± s.d. n = 3. Fig. S10 Colon targeting of PEG-CNPs (1.5 mg/kg) in colon tissues. Data represent means ± s.d. n = 3. ***P < 0.001 vs. control group. Fig. S11 Representative fluorescence images of HT-29 cells (a) and RAW 264.7 cells (b) after incubation with FITC-PEG-CNPs for 24 h. Scale bars: 50 μm. Fig. S12 Morphology of RAW 264.7 cells after treated with LPS (-/+) (100 ng/ml) and PEG-CNPs. Table S1 Sequences of the primers used for qRT-PCR. Table S2 The evaluation criteria of disease activity index (DAI) of mice. Table S3 Pharmacokinetic parameters of PEG-CNPs. Data represent means ± s.d. n = 3. [file 40824_2023_412_MOESM1_ESM.docx]

*Supporting Information*

**Restoration of dysregulated intestinal barrier and inflammatory regulation through synergistically ameliorating hypoxia and scavenging reactive oxygen species using ceria nanozymes in ulcerative colitis**

Ying Zhang^1,2^, Hengyu Lei^2^, Pengchong Wang^3^, Qinyuan Zhou^2^, Jie Yu^2^, Xue Leng^2^, Ruirui Ma^2^, Danyang Wang^2^, Kai Dong^2*^, Jianfeng Xing^2*^, Yalin Dong^1*^.

^1^Department of Pharmacy, The First Affiliated Hospital of Xi’an Jiaotong University, Xi’an, Shaanxi, China.

^2^Department of Pharmaceutics, School of Pharmacy, Xi’an Jiaotong University, Xi’an, Shaanxi, China.

^3^Department of Pharmacy, Shaanxi Provincial People's Hospital, Xi’an, Shaanxi, China.

***Corresponding author: Kai Dong, Ph.D.**

Department of Pharmacy, Xi’an Jiaotong University, Xi’an 710061, China.

Phone: +86-29-82655139

Email: dongkai120@mail.xjtu.edu.cn

***Corresponding author: Jianfeng Xing, Ph.D.**

Department of Pharmacy, Xi’an Jiaotong University, Xi’an 710061, China.

Phone: +86-29-82655139

Email: xajdxjf@mail.xjtu.edu.cn

***Corresponding author: Yalin Dong, Ph.D.**

Department of Pharmacy, The First Affiliated Hospital of Xi’an Jiaotong University, Xi’an 710061, China.

Phone: +86-29-85323241

Email: dongyalin@mail.xjtu.edu.cn

**
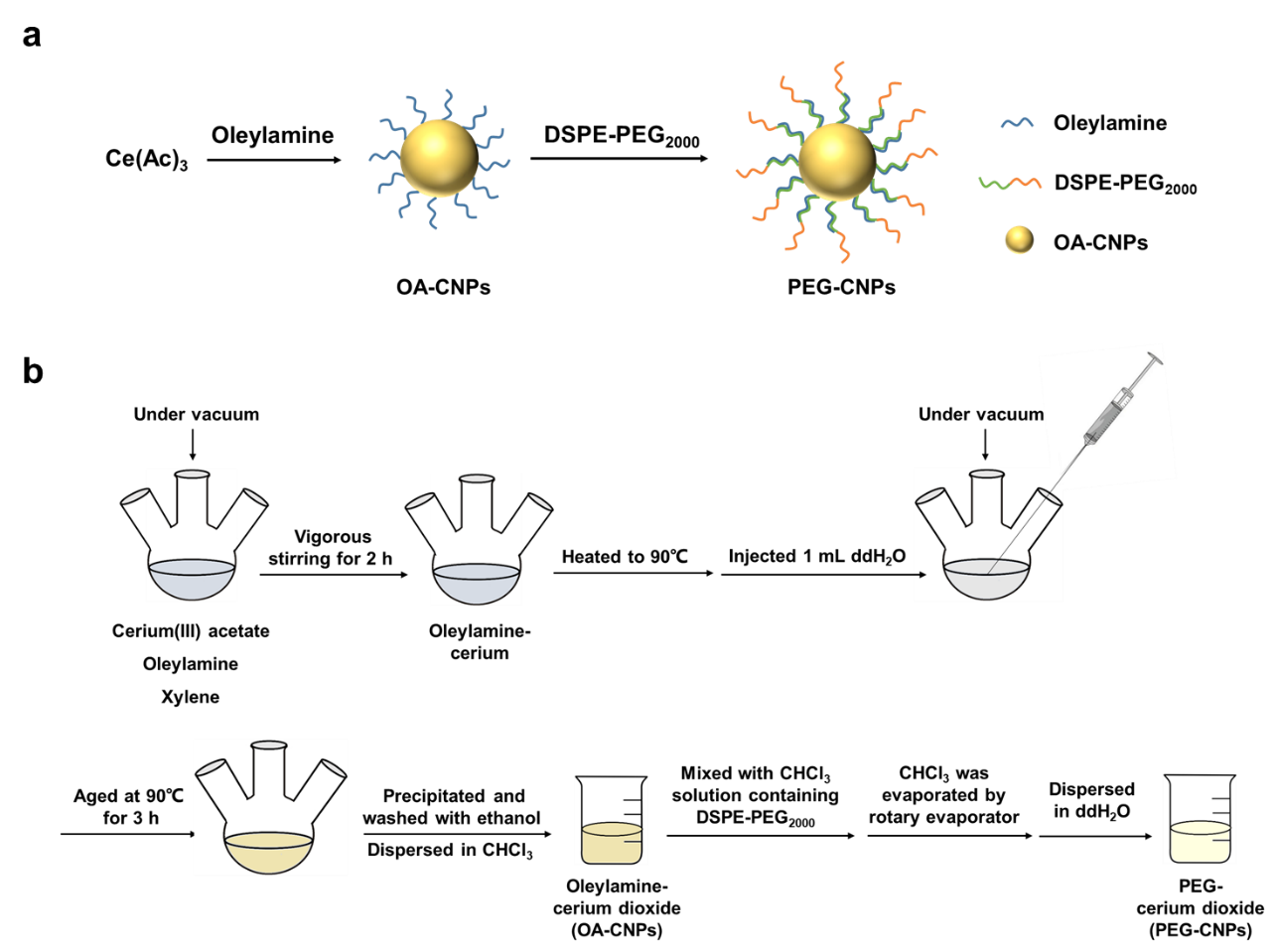
**

Fig. S1 Scheme of PEG-CNPs preparation via a modified reverse micelle method. (a) Synthetic schematic for PEG-CNPs preparation. (b) Detailed process for PEG-CNPs preparation.


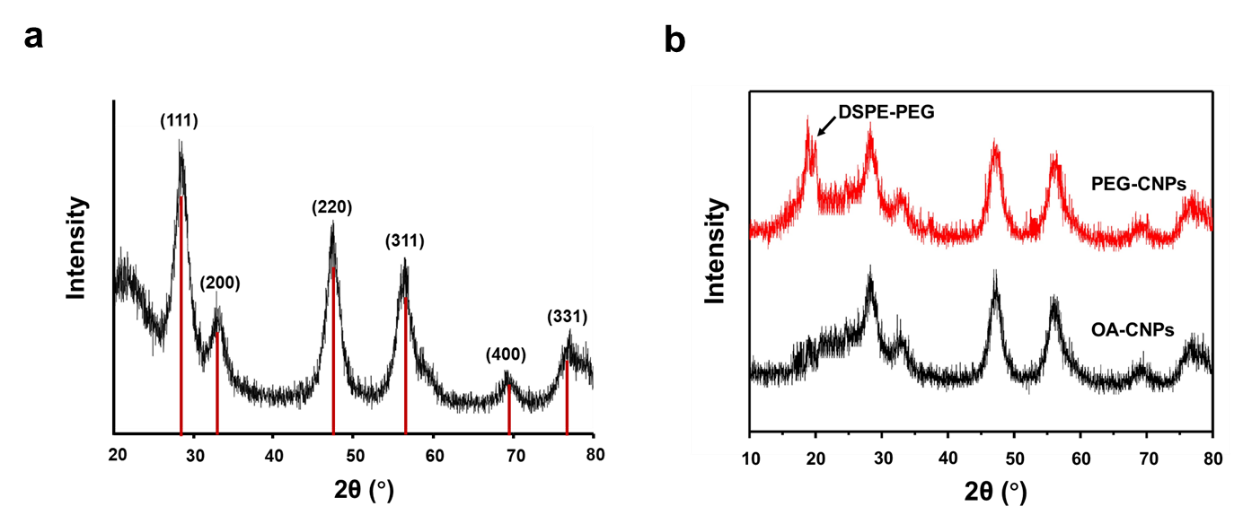


Fig. S2 The XRD pattern shows the structure of OA-CNPs (a) and PEG-CNPs (b).


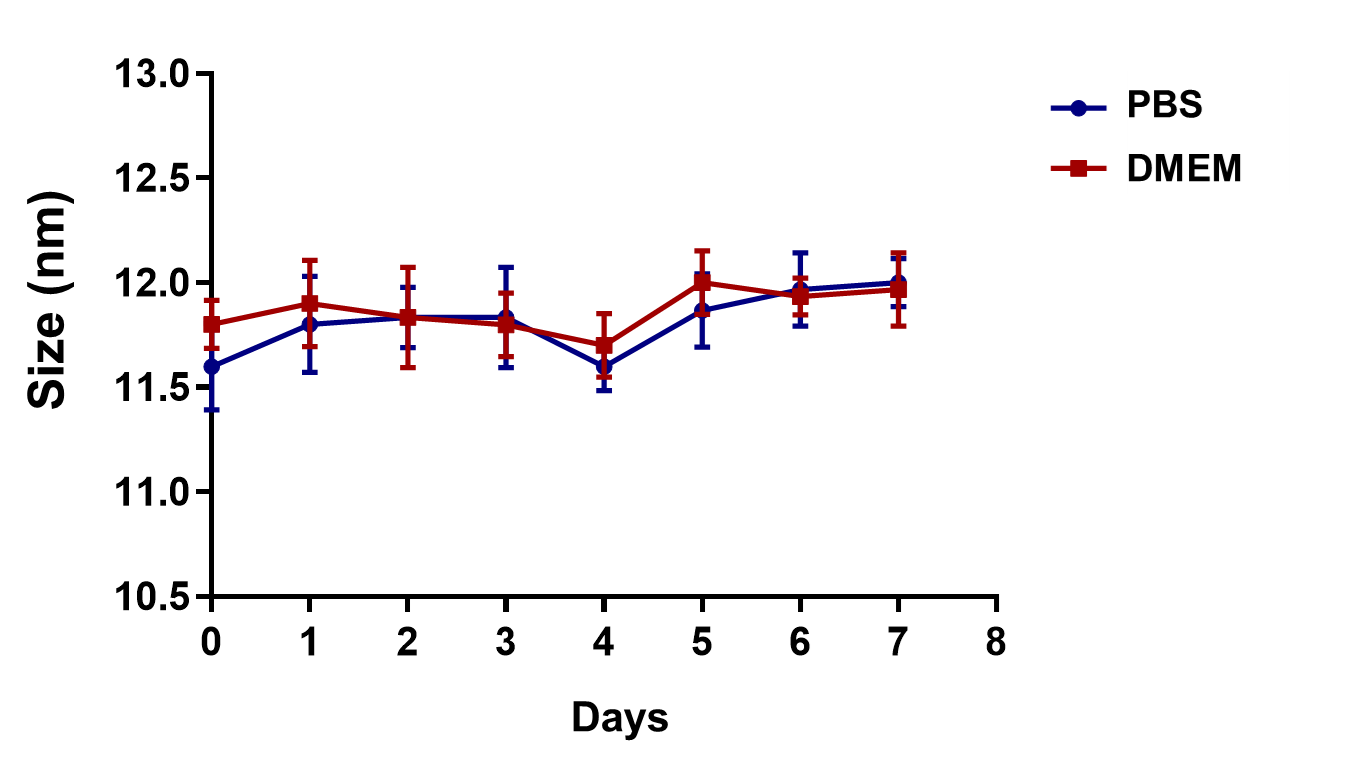


Fig. S3 The colloidal stability of PEG-CNPs in PBS or DMEM containing 10% fetal bovine serum (FBS) within 7 days. Data represent means ± s.d. n = 3.


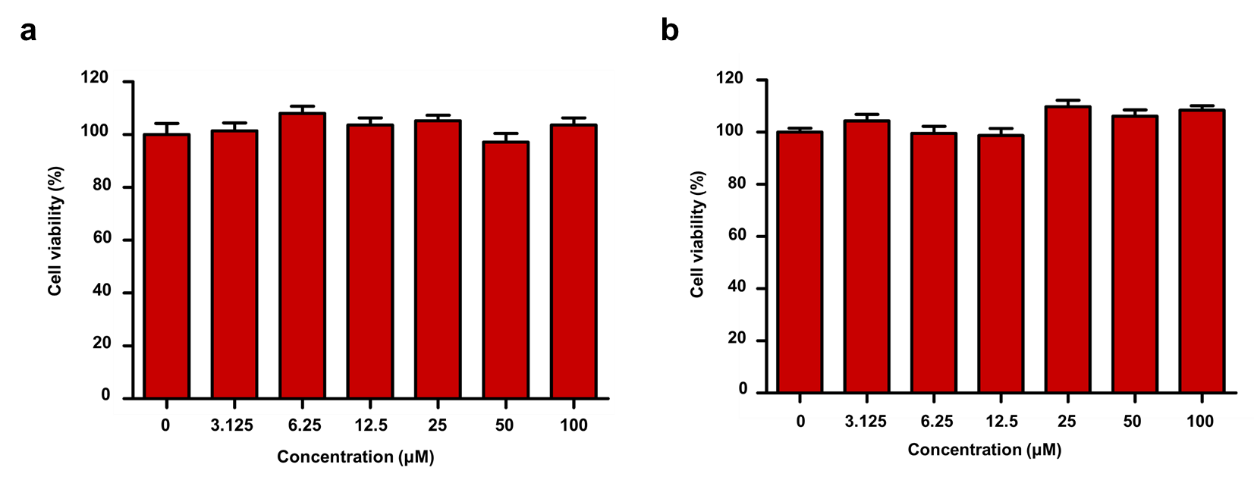


Fig. S4 Cytotoxicity of PEG-CNPs in (a) RAW 264.7 cells and (b) HT-29 cells. Cell viability was evaluated at 24 h of exposure to PEG-CNPs. Data represent means ± s.d. n = 6.


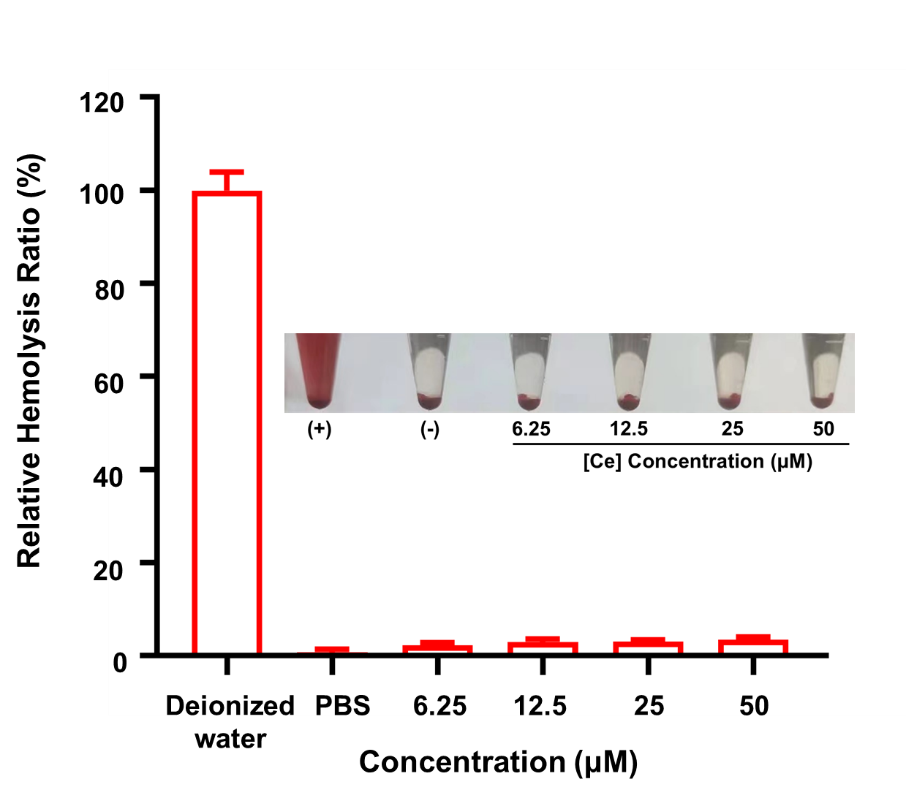


Fig. S5 Photographs of red blood cells and the hemolytic activity of PEG-CNPs. Data represent means ± s.d. n = 3.


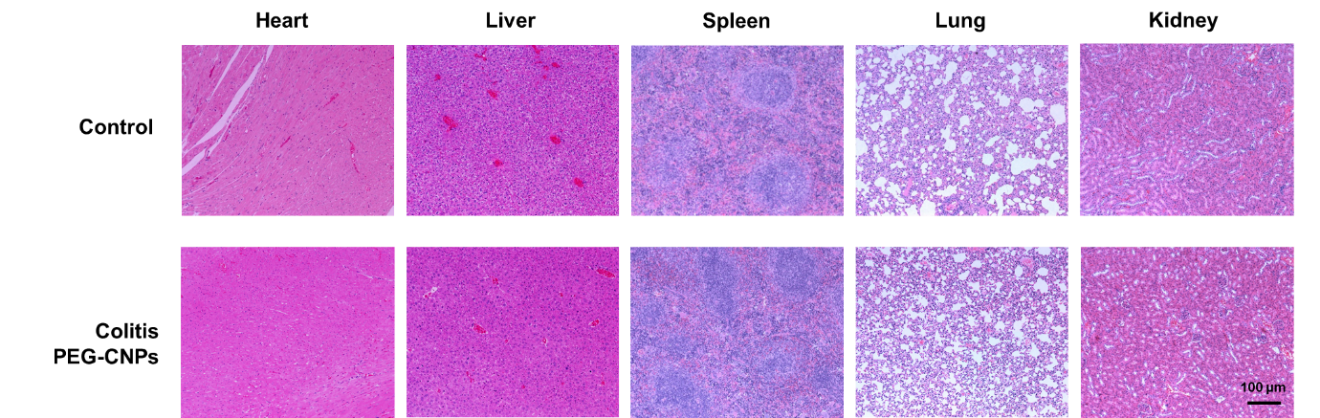


Fig. S6 Representative H&E staining of major organs from colitis mice at day 5 after 1.5 mg/kg PEG-CNPs treatment.


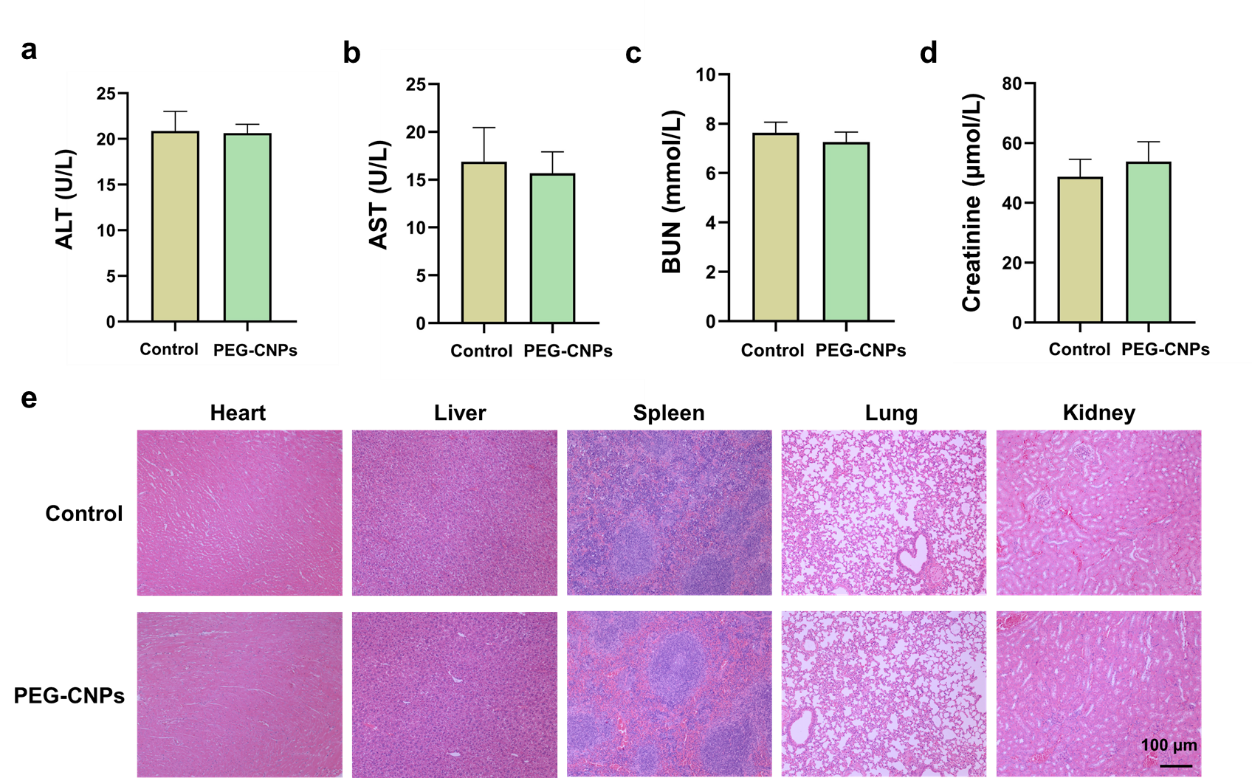


Fig. S7 Long-term toxicity of PEG-CNPs in healthy mice. Blood biochemistry analyses of ALT (a), AST (b), BUN (c) and creatinine (d) of mice at day 21 after 1.5 mg/kg PEG-CNPs treatment. (e) Representative H&E staining of major organs from mice. Data represent means ± s.d. n = 6.


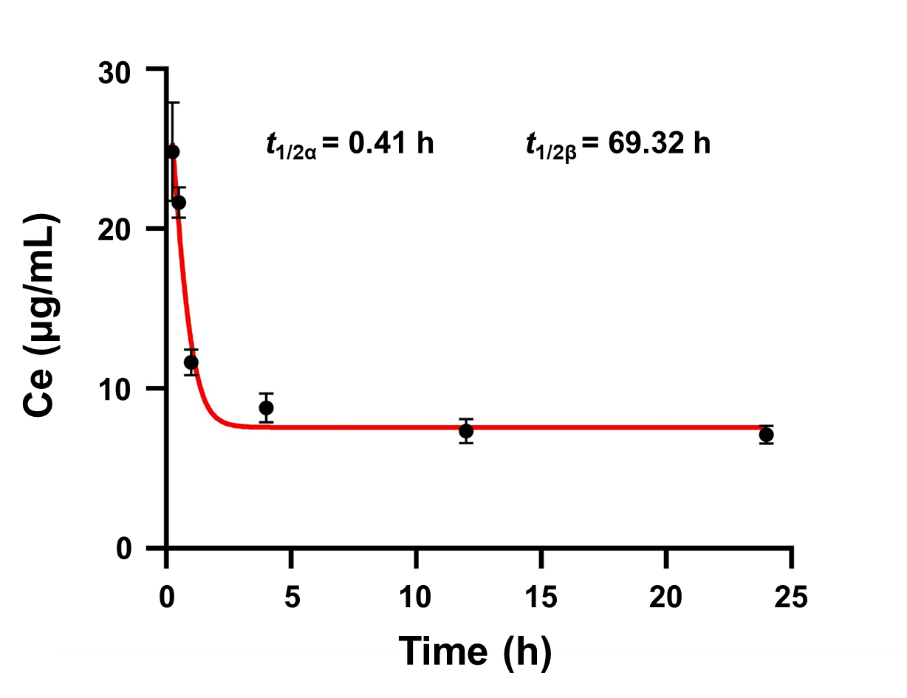


Fig. S8 *In vivo* pharmacokinetic curves of PEG-CNPs (1.5 mg/kg). Data represent means ± s.d. n = 3.


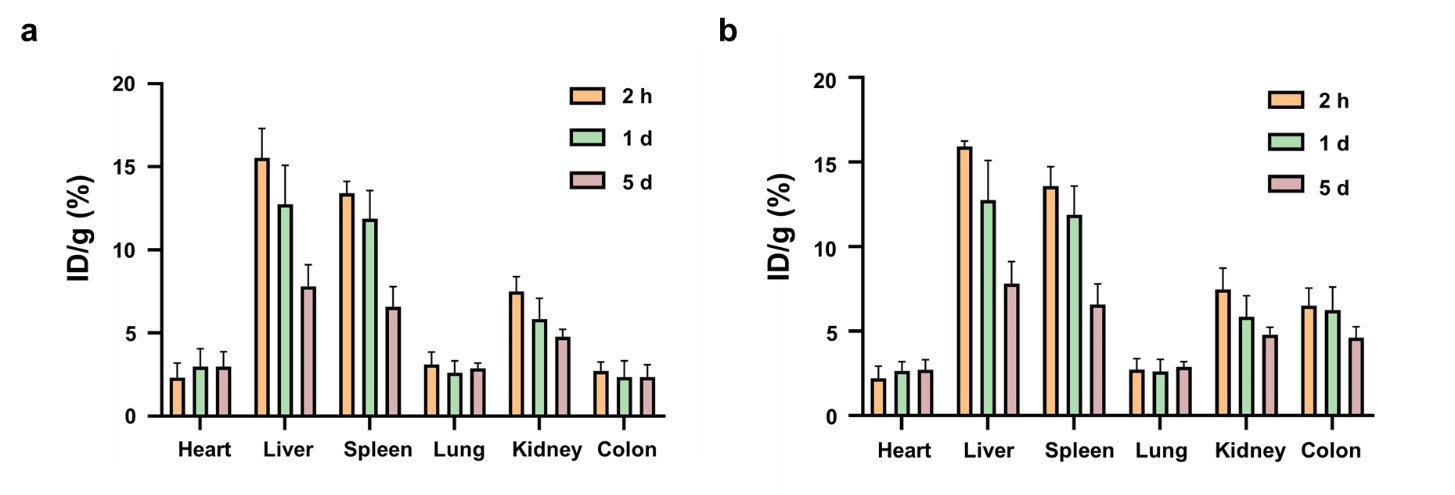


Fig. S9 Biodistribution of Ce element at different time points after intravenous injection of PEG-CNPs (1.5 mg/kg) in healthy mice (a) and colitis mice (b). Data represent means ± s.d. n = 3.


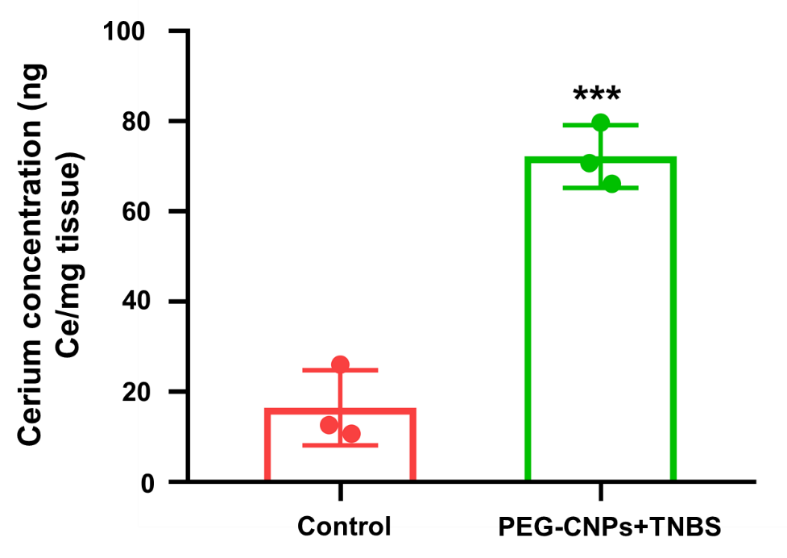


Fig. S10 Colon targeting of PEG-CNPs (1.5 mg/kg) in colon tissues. Data represent means ± s.d. n = 3. **^***^***P* < 0.001 vs. control group.


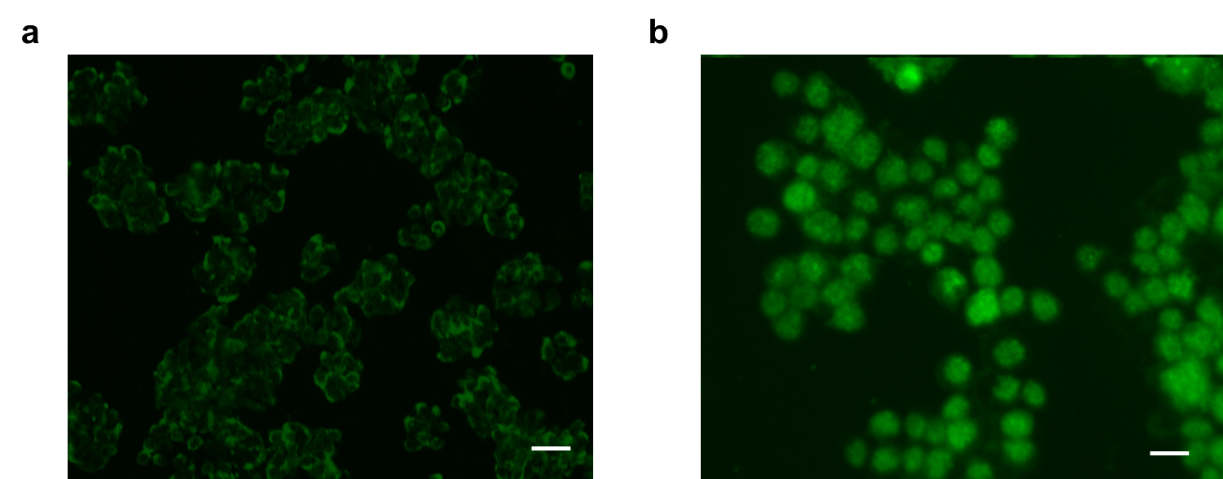


Fig. S11 Representative fluorescence images of HT-29 cells (a) and RAW 264.7 cells (b) after incubation with FITC-PEG-CNPs for 24 h. Scale bars: 50 μm.


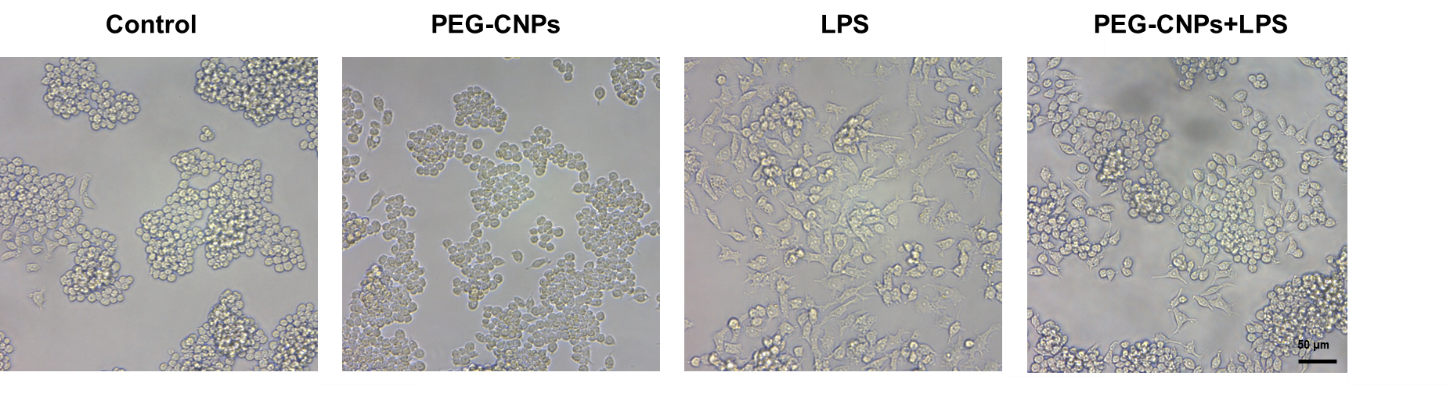


Fig. S12 Morphology of RAW 264.7 cells after treated with LPS (-/+) (100 ng/ml) and PEG-CNPs.

Table S1 Sequences of the primers used for qRT-PCR.

| Serial number | Primer name | Forward sequences | Reverse sequences |
| --- | --- | --- | --- |
| 1 | Human *HO-1* | 5-CTCTCTTCTCTTGGGCCTCTAA-3 | 5-TGTCAGGTATCTCCCTCCATTC-3 |
| 2 | Human *Nqo1* | 5-GAGAAGAGCCCTGATTGTACTG-3 | 5-ACCTCCCATCCTCTCTTCTT-3 |
| 3 | Human *Gpx1* | 5-TGCGAAGTGAATGGTGAGAA-3 | 5-CACCGGAGACCAAATGATGTA-3 |
| 4 | Human *NOX2* | 5-GGCCATGGAGTGGACTTAAA-3 | 5-GATACACCTCTCCACCAATGAC-3 |
| 5 | Human *Cyp2e1* | 5-CACAGCCAAGAACCCATGTA-3 | 5-CACAGCCAAGAACCCATGTA-3 |
| 6 | Human *Nrf2* | 5-CTCCGTGGAGTCTTCCATTTAC-3 | 5-GCACTATCTAGCTCCTCCATTTC-3 |
| 7 | Human *Keap1* | 5-CTCTGAGCCCTGAACAGTTATT-3 | 5-CTGTGTGCCTACTCCATTCTT-3 |
| 8 | Human *DJ-1* | 5-GCAGTGTAGCCGTGATGTAA-3 | 5-CACCATAGGCGACTCAGATAAA-3 |
| 9 | Mouse *IL-1β* | 5-CTCCACCTCAATGGACAGAATATC-3 | 5-GGGTGTGCCGTCTTTCATTA-3 |
| 10 | Mouse *TNF-α* | 5-CGATGGGTTGTACCTTGTCTAC-3 | 5-GAGGTTGACTTTCTCCTGGTATG-3 |
| 11 | Mouse *HIF-1α* | 5-CCACCTATGACCTGCTTGGT-3 | 5-TATCCAGGCTGTGTCGACTG-3 |
| 12 | Human *GAPDH* | 5-GGTGTGAACCATGAGAAGTATGA-3 | 5-GAGTCCTTCCACGATACCAAAG-3 |
| 13 | Mouse *GAPDH* | 5-TCAACAGCAACTCCCACTCTTCCA-3 | 5-ACCCTGTTGCTGTAGCCGTATTCA-3 |
| 14 | Human Si*Nrf2* | 5-CCUGAAAGCACAGCAGAAUTT-3 | 5-AUUCUGCUGUGCUUUCAGGTT-3 |

Table S2 The evaluation criteria of disease activity index (DAI) of mice.

| Weight loss | Stool consistency | Fecal occult blood | Score |
| --- | --- | --- | --- |
| none | well-formed pellets | normal | 0 |
| 1%-5% | loose stools | Occult blood + | 1 |
| 5%-10% | diarrhea | Occult blood + + | 2 |
| 10%-15% | diarrhea | Occult blood + + + | 3 |
| > 15% | diarrhea | visible gross bleeding | 4 |

Table S3 Pharmacokinetic parameters of PEG-CNPs. Data represent means ± s.d. n = 3.

| **Parameters** | **Unit** | **Value** |
| --- | --- | --- |
| *t*_1/2α_ | h | 0.414 ± 0.047 |
| *t*_1/2β_ | h | 69.265 ± 0.086 |
| V | L/kg | 0.044 ± 0.006 |
| CL | L/h/kg | 0.004 ± 0.001 |
| AUC_0-24_ | mg/L·h | 207.574 ± 9.000 |
| AUC_0-∞_ | mg/L·h | 415.003 ± 66.127 |
| *k*_10_ | 1/h | 0.086 ± 0.025 |
| *k*_12_ | 1/h | 1.331 ± 0.424 |
| *k*_21_ | 1/h | 0.413 ± 0.019 |
